# Density-functional tight-binding: basic concepts and applications to molecules and clusters

**DOI:** 10.1080/23746149.2019.1710252

**Published:** 2020-02-18

**Authors:** Fernand Spiegelman, Nathalie Tarrat, Jérôme Cuny, Leo Dontot, Evgeny Posenitskiy, Carles Martí, Aude Simon, Mathias Rapacioli

**Affiliations:** a Laboratoire de Chimie et Physique Quantiques LCPQ/IRSAMC, UMR5626, Université de Toulouse (UPS)and CNRS, Toulouse, France; b CEMES, Université de Toulouse (UPS), CNRS, UPR8011, Toulouse, Toulouse, France; c Laboratoire Collisions Agrégats et Réactivité LCAR/IRSAMC, UMR5589, Université de Toulouse (UPS) and CNRS, Toulouse, France; d Laboratoire de Chimie, UMR5182, Ecole Normale Supérieure de Lyon, Université de Lyon and CNRS, Lyon, France

**Keywords:** DFTB, electronic structure, simulation, molecules, clusters

## Abstract

The scope of this article is to present an overview of the Density Functional based Tight Binding (DFTB) method and its applications. The paper introduces the basics of DFTB and its standard formulation up to second order. It also addresses methodological developments such as third order expansion, inclusion of non-covalent interactions, schemes to solve the self-interaction error, implementation of long-range short-range separation, treatment of excited states *via* the time-dependent DFTB scheme, inclusion of DFTB in hybrid high-level/low level schemes (DFT/DFTB or DFTB/MM), fragment decomposition of large systems, large scale potential energy landscape exploration with molecular dynamics in ground or excited states, non-adiabatic dynamics. A number of applications are reviewed, focusing on -(i)- the variety of systems that have been studied such as small molecules, large molecules and biomolecules, bare orfunctionalized clusters, supported or embedded systems, and -(ii)- properties and processes, such as vibrational spectroscopy, collisions, fragmentation, thermodynamics or non-adiabatic dynamics. Finally outlines and perspectives are given.

## 1 Introduction

Since the demonstration by Hohenberg and Kohn [1] of the theoretical grounding of the Density Functional Theory (DFT) [2–4], stating that the energy of any electronic system is a universal functional of the density *ρ* and the proposal of the Kohn-Sham scheme [5] to find the density, DFT has proved ubiquitous in the theoretical description of electronic system properties of atoms, molecules and condensed matter [6,7]. It has become a choice tool for atomic-scale simulations in Chemistry and Material Science [6–8]. In the Kohn-Sham formulation, the energy of the actual many interacting electrons system is shown to be equivalent to that of a fictitious system of independent electrons within an effective potential involving the interaction with the nuclei (and possibly external potentials) complemented by the electron-electron Coulomb interaction and the exchange-correlation functional *E*_*xc*_[*ρ*]

(1) $$E\left[\rho \right]=\sum_{k}n_{k}\langle \varphi_{k}\left|-\frac{1}{2}\Delta \right|\varphi_{k}\rangle +V_{\text{ext}}\left[\rho \right]+\frac{1}{2}\int \frac{\rho \left(\text{r}\right)\rho \left({\text{r}}^{\prime }\right)}{\left|{\text{r}}^{\prime }-\text{r}\right|}{\text{d}}^{3}\text{r}{\text{d}}^{3}{\text{r}}^{\prime }+E_{\text{xc}}\left[\rho \right]+\frac{1}{2}\sum_{a,b}\frac{Z_{a}Z_{b}}{\left|{\text{R}}_{a}-{\text{R}}_{b}\right|}$$

The first term is the kinetic energy of independent electrons in orbitals *φ*_*k*_ weighted by their occupation numbers. V_*ext*_ is the functional contribution associated with the external potential *v*_*ext*_. Applying the variational theorem, the resolution is obtained in terms of the mean-field type Kohn-Sham (KS) equation

(2) $$\left(-\frac{1}{2}\Delta +v_{ext}\left(\text{r}\right)+\int \frac{\rho \left({\text{r}}^{\prime }\right)}{\left|{\text{r}}^{\prime }-\text{r}\right|}{\text{d}}^{3}{\text{r}}^{\prime }+\frac{\delta E_{xc}}{\delta \rho \left(\text{r}\right)}\right)\varphi_{k}=\epsilon_{k}\varphi_{k}$$

The left hand side of the above equation is the Kohn-Sham operator *H* ^*KS*^ = $\frac{\delta E}{\delta \rho }$ consisting of the sum of the kinetic contribution and the Kohn-Sham potential *v*_*KS*_

(3) $$v_{KS}\left[\rho \right]=v_{ext}\left(\text{r}\right)+\int \frac{\rho \left({\text{r}}^{\prime }\right)}{\left|{\text{r}}^{\prime }-\text{r}\right|}{\text{d}}^{3}{\text{r}}^{\prime }+\frac{\delta E_{xc}}{\delta \rho \left(\text{r}\right)}$$

The density (normalized to the number of electrons) is obtained from the individual orbitals

(4) $$\rho \left(\text{r}\right)=\sum_{k}n_{k}{\left|\varphi_{k}\left(\text{r}\right)\right|}^{2}$$

The Kohn-Sham operator depends on the orbitals *via* the density and must hence be solved self-consistently. While the Kohn-Sham equation is mathematically very similar to the Hartree-Fock equation, a major difference lies in the fact that it formally incorporates the electron-electron correlation. On the opposite, the Hartree-Fock energy must be complemented by a wavefunction type many-body correlation contribution based on multiconfigurational schemes with a generally unfavorable dependence to the number of electrons. Conversely to many-body wavefunctions which are functions of coordinates in space *R* ^3*N*^, the electronic density is only a function of variables in *R* ^3^. Hence, the resolution of the KS equation is much simpler and computationally much more efficient than Configuration Interaction type schemes, which explains the success of DFT. Using linear scaling algorithms and High Performance Computing systems, DFT is now able to deal with a few thousands of atoms and a few tens of thousands of electrons at least for a single geometry. Of course, the main theoretical handicap of DFT is that the exchange-correlation functional remains unknown. This brings various drawbacks in many applications of DFT such as the self-interaction error (SIE) [4,9–13], and consequent inherent failures like improper description of the charge localization in extended compounds, ill-behaved dissociation or an incorrect energy derivative with the number of electrons. The account of dispersion forces is also problematic in standard DFT functionals. This situation has led to the proposal of a forest of functionals, some of them taking advantage of theoretical grounding, other empirically determined over reference training sets. This has sometimes questioned the practice of DFT as a first principle theory. Many progresses are currently done to design improved functionals, in particular based around the concept of long-range correction (LC) through a short-range/long-range separation [4,14–17] and its account through double hybrid functionals [17]. Correction of SIE and improvements of functionals are also major challenges in the representation of excited states *via* the time-dependent version of DFT (TD-DFT), in particular to properly describe Rydberg states or charge transfer excitations.

Despite the favorable computational adaptation of DFT and dedicated progress to achieve linear scaling, there is always a need from the computational point of view for even more efficient techniques. This is the case if one aims at modelling larger systems in the nanoscale domain for instance or running Molecular Dynamics (MD) or Monte Carlo (MC) simulations for medium size systems with the scope of reaching statistical convergence, which requires calculations of energies and energy gradients that must be repeated up to 10^6^-10^8^ times or even more. The development of approximate schemes, still treating electrons quantum-mechanically, has always been a challenge since the early years of quantum chemistry. There have been essentially two ways for designing such schemes. One is offered by most of the approximate single electron descriptions, which start with very simple elements and can be further complexified in a bottom-up strategy.

**Figure 1:**
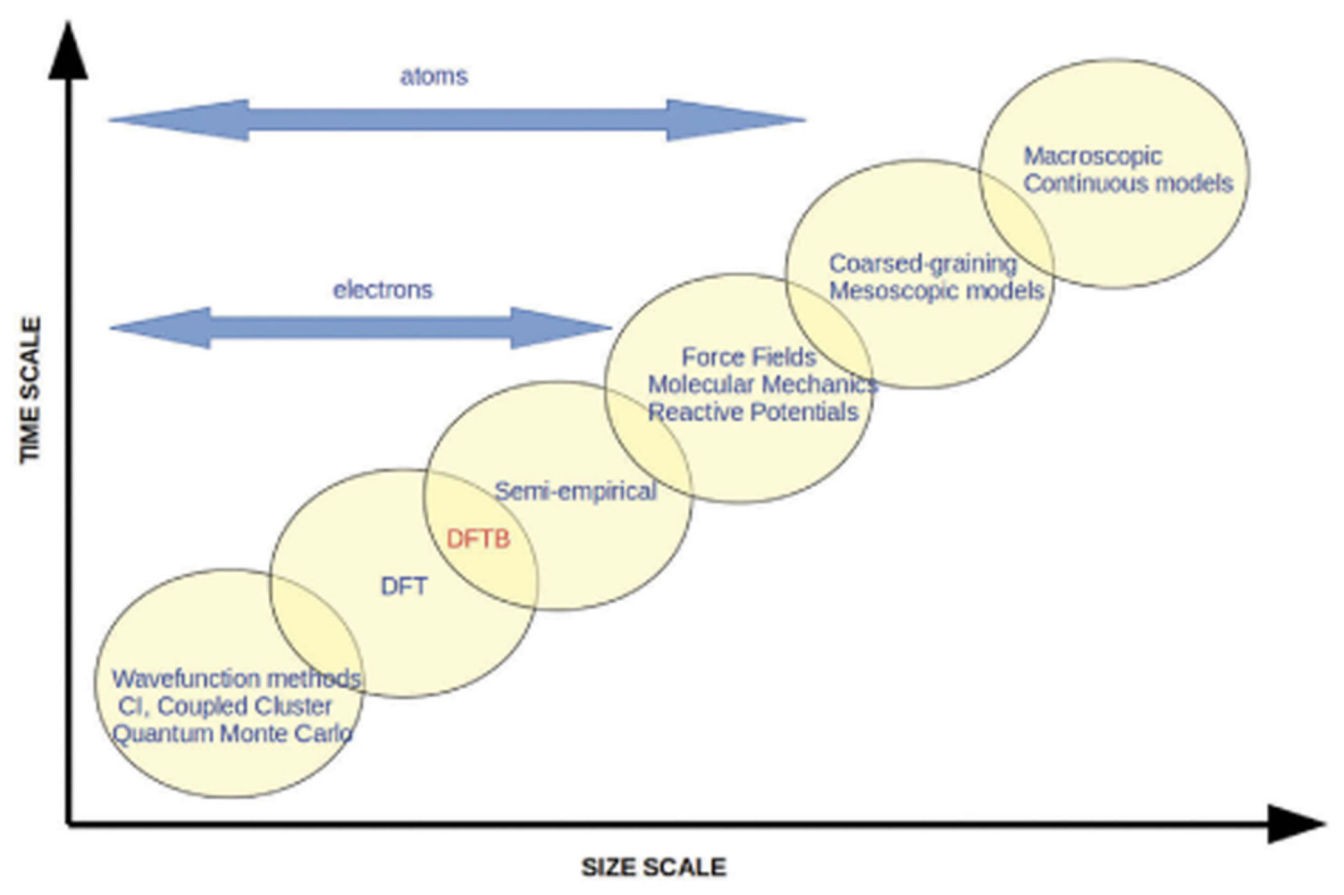
Size and time scales of simulation methods used in chemistry and material science.

The second one, more recent and efficient, tends to be theoretically derived in a top-down approximation scheme, from well established mean-field theories, formerly Hartree-Fock and now DFT. It is in this last scheme that the Density Functional based Tight Binding (DFTB) formalism [18–20] has been developed over the two-three decades, now described in a number of review [20–24] or introductory [25] articles. The position of DFTB among other simulation methods in terms of size and simulated time scales is shown in Figure 1. The scope of the present article is (i) to provide an overview of the principles and advances of DFTB in the domain of electronic structure and molecular simulation and (ii) to illustrate applications to molecules, clusters and nanoparticles.

Section 2 introduces the basic formalism and approximations of DFTB. Section 3 describes developments and extensions such as description of non-covalent forces, improvement of electrostatics, inclusion of DFTB in hybrid methods or determination of electronic excited states. The use of DFTB in large scale simulations (global optimization, dynamics in ground and excited states or thermodynamics) is also commented. After reporting the accuracy of DFTB on small molecules, section 4 overviews applications to more involved classes of systems such as biomolecules, bare or functionalized clusters and nanoparticles, or supported/embedded systems. Note that the number of articles within the DFTB framework is now too large to allow for a fully exhaustive account in the present review article. Hence, the application sections should only be considered as an attempt to provide representative DFTB applications to various fields of chemistry and molecular physics. Finally, outlines and perspectives are given in the last section. Throughout the paper, we will in general use *a*, *b*, *c*, *d* to label atoms, greek letters *μ*, *ν*, *λ*, *τ* … to label atomic orbitals, *i*, *j*, *k*, *l* … for molecular orbitals, and capital letters *A*, *B*, *C* … to address fragment systems. **R**, **R**_*a*_, and **r** will label global nuclei coordinates, nuclei coordinates of atom *a* and electronic coordinates, respectively.

## 2 The density-functional based tight-binding approach: basic concepts

### 2.1 A brief overview of tight-binding theories

Prior to describe the principles of the DFTB method in details, we provide in this subsection a brief general framework for Tight-Binding (TB) theories. Simplified quantum methods for electronic structure rely on several general approximations. A first one concerns the restriction of the Hamiltonian to a subclass of electrons directly involved in the electronic properties of interest. Consideration of the valence electrons only is also related to the physics and chemistry underlying frozen cores and pseudopotential schemes in *ab initio* calculations. In general, model valence Hamiltonians are defined in linear combination of atomic orbital (LCAO)-type basis sets, so-called minimal in the sense that each valence orbital *μ* of atom *a* is defined by a single atomic function *ϕ_aμ_*. This is a basic assumption of early quantum semi-empirical methods, as featured by the Hückel [26] or extended-Hückel Hamiltonians [27–30] of quantum chemistry or the tightbinding equivalent in solid state [31–33] and surface physics [34,35] corresponding to one-electron pictures. Restriction to the valence space is also the basis of semi-empirical, multi- or mono-configurational approximations of quantum chemistry such as CNDO [36], MNDO [37], AM1 [38] and PM3 [39]. It remains the basis of the modern tight-binding versions [21,40]. In all these schemes, the basis set is implicit and the Hamiltonian is defined in the matrix form. Transferability and flexibility are accounted for by the dependence of the matrix elements upon geometry [41].

A generic electronic TB Hamiltonian is defined by its matrix elements

(5) $$H_{a\mu ,bv}=\langle \phi_{a\mu }|\tilde{H}|\phi_{bv}\rangle$$

expressed in the minimal LCAO representation. The diagonal elements have the meaning of effective single-electron atomic energy levels associated with the valence shell atomic orbitals, possibly screened by an effective potential *Ṽ* not necessarily explicited:

(6) $$H_{a\mu ,a\mu }=\langle \phi_{a\mu }\left|-\frac{\Delta }{2}+\tilde{V}\right|\phi_{a\mu }\rangle =\varepsilon_{a\mu }$$

while the interatomic off-diagonal elements between orbitals of different atoms (*a* ≠ *b*), called hopping integrals, describe electron delocalization primarily induced by the (screened) kinetic energy operator

(7) $$H_{a\mu ,bv}=\langle \phi_{a\mu }\left|-\frac{\Delta }{2}+\tilde{V}\right|\phi_{bv}\rangle$$

The on-site off diagonal elements are generally zero.

In a LCAO non-orthogonal basis, the tight-binding eigenvalue problem is solved for the orbitals *φ*_*k*_ and energies *ε*_*k*_

(8) $$\varphi_{k}=\sum_{a\mu }c_{a\mu }^{k}\phi_{a\mu }$$

*via* the set of secular equations

(9) $$\sum_{bv}\left(H_{a\mu ,bv}-\varepsilon_{k}S_{a\mu ,bv}\right)c_{bv}^{k}=0,\forall a\mu$$

If the atomic basis functions are supposed to be orthogonal, thus giving rise to the orthogonal tight-binding scheme, the one-electron levels and orbitals are simply obtained by diagonalizing the effective Hamiltonian matrix.

Labelling *ρ*_*aμ,bν*_ the one-particle density matrix elements by the elements of the one-particle density matrix *ρ̂*, the sum of the valence electrons energies is

(10) $$\sum_{k}n_{k}\varepsilon_{k}=\sum_{k}\sum_{a\mu ,bv}n_{k}c_{a\mu }^{k*}c_{bv}^{k}H_{a\mu ,bv}=\sum_{a\mu ,bv}\rho_{a\mu ,bv}H_{a\mu ,bv}$$

Finally, the total TB energy can be cast under the very general form, consistent with DFT:

(11) $$E\left[\rho \right]=V_{\text{rep}}\left(\text{R}\right)+\sum_{k}n_{k}\varepsilon_{k}+G\left[\rho \right]$$

where *V*_rep_ (**R**) essentially describes the short-range repulsion of the ionic cores, the sum of the single electron energies defines the band energy and the functional contribution of the density *G*[*ρ*] provides an account of all residual contributions, namely the exchange and correlation energies (in particular the dispersion contribution) that are not included in the effective band contribution, as well as the double-counting corrections (the most important being the double counting of Coulomb terms when relevant).

In the simplest version with no electrostatics and no self-consistency included, *Ṽ* is supposed to account for electron screening. In the case of ionic or iono-covalent systems or systems with significant charge fluctuations, interactions between on-site charges can be taken into account, either perturbatively [42–44] or self-consistently [21,40,45–51]. Tightbinding methods may also be considered according to the origin of their parametrization: either semi-empirical tight-binding, where simple functional forms are used for the matrix elements fitted to reproduce *ab initio* or experimental data, or *ab initio* tight-binding, where the formalism, functions and inputs are fully derived from first principles references [47].

### 2.2 From DFT to DFTB

The basic idea of DFTB consists in an expansion of the density *ρ*(*r*) = *ρ*_0_(*r*) + *δρ*(*r*) around a reference density *ρ*_0_(*r*)

(12) $$\begin{array}{l} E\left[\rho \left(r\right)\right]=E\left(\left[\rho_{0}\left(\text{r}\right)\right]\right)+\int {\frac{\delta E\left[\rho \left(\text{r}\right)\right]}{\delta \rho \left(\text{r}\right)}|}_{\rho_{0}}\delta \rho \left(\text{r}\right)+\frac{1}{2}{\iint \frac{\delta^{2}E\left[\rho \left(\text{r}\right)\right]}{\delta \rho \left(\text{r}\right)\delta \rho \left({\text{r}}^{\prime }\right)}|}_{\rho_{0}}\delta \rho \left(\text{r}\right)\delta \rho \left({\text{r}}^{\prime }\right)\ldots \\ +\frac{1}{p!}{\iint \ldots \int \frac{\delta E\left[\rho \left({\text{r}}^{\prime }\right)\right]}{\delta \rho \left(\text{r}\right)\delta \rho \left({\text{r}}^{\prime }\right)\ldots \delta \rho \left({\text{r}}^{\left(\text{p}\right)}\right)}|}_{\rho_{0}}\delta \rho \left(\text{r}\right)\delta \rho \left({\text{r}}^{\prime \prime }\right)\ldots \delta \rho \left({\text{r}}^{\left(\text{p}\right)}\right)+\ldots \end{array}$$

In current DFTB schemes, the superposed density of the atoms (isolated or in a confined potential) is taken as starting point *ρ*_0_. Collecting the terms which depend on *ρ*_0_ only in a so-called repulsive energy contribution, one has

(13) $$E^{rep}=E\left(\rho_{0}\right)-\int {\frac{\delta E}{\delta \rho }|}_{\rho_{0}}\rho_{0}\left(\text{r}\right)d\text{r}$$

Using the expression of the Kohn-Sham operator, the terms depending on *ρ* only provide the so-called band-energy, which was the basis of the initial version of DFTB or DFTB1 [18] (including the above repulsion energy).

(14) $$E^{\left(1\right)}={\int \frac{\delta E}{\delta \rho }|}_{\rho_{0}}\rho \left(\text{r}\right)d\text{r}$$

The second order dependence upon density fluctuation of the Coulomb and of the exchange-correlation energy only appears in the second order term, namely

(15) $$E^{\left(2\right)}=\frac{1}{2}\iint \left(\frac{1}{\left|\text{r}-{\text{r}}^{\prime }\right|}+{\frac{\delta^{2}E_{xc}}{\delta \rho \left(\text{r}\right)\delta \rho \left({\text{r}}^{\prime }\right)}|}_{\rho_{0}}\right)\delta \rho \left(\text{r}\right)\delta \rho \left({\text{r}}^{\prime }\right)d\text{r}d{\text{r}}^{\prime }$$

This provides the second order or DFTB2 expansion, namely

(16) $$E^{DFTB2}=E^{rep}+\sum_{i}n_{i}\langle \varphi_{i}\left|H_{0}^{KS}\right|\varphi_{i}\rangle >+\frac{1}{2}\iint \left(\frac{1}{\left|\text{r}-{\text{r}}^{\prime }\right|}+{\frac{\delta^{2}E_{xc}}{\delta \rho (\text{r})\delta \rho \left({\text{r}}^{\prime }\right)}|}_{\rho_{0}}\right)\delta \rho \left(\text{r}\right)\delta \rho \left({\text{r}}^{\prime }\right)d\text{r}d{\text{r}}^{\prime }$$

which is the most widely spread DFTB scheme, also called self-consistent charge DFTB (SCC-DFTB) [19,20]. The next step consists in expressing the molecular orbitals as linear combinations of atomic orbitals, consequently defining the matrix elements of the Kohn-Sham operator for the reference density

(17) $$H_{a\mu ,bv}^{0}=\langle \phi_{a\mu }\left|H_{0}^{KS}\right|\phi_{bv}\rangle$$

Another approximation consists in replacing the 3D continuous electronic density by a set of discretized atomic electron populations. Assuming a nonpolar expansion of the density fluctuation *δρ*(*r*) over the atomic centers

(18) $$\delta \rho \left(\text{r}\right)=\sum_{a}\Delta q_{a}F_{0}\left(\text{r}-{\text{R}}_{a}\right)$$

the electrostatic situation is described by atomic charges fluctuations Δ*q*_*a*_ with respect to the atomic neutral references. In the standard versions of DFTB, Mulliken’s charges are used [52]. One should note here that atomic charges are not observables and their definition is arbitrary (see below section 3.1).

One can then introduce the two-electron integrals *γ_ab_* as

(19) $$\gamma_{ab}=\iint \left(\frac{1}{\left|\text{r}-{\text{r}}^{\prime }\right|}+{\frac{\delta^{2}E_{xc}}{\delta \rho (\text{r})\delta \rho \left({\text{r}}^{\prime }\right)}|}_{\rho_{0}}\right)F_{0}\left(\text{r}-{\text{R}}_{a}\right)F_{0}\left({\text{r}}^{\prime }-{\text{R}}_{b}\right)d\text{r}d{\text{r}}^{\prime }$$

and the total DFTB2 energy reads

(20) $$E=E^{rep}+\sum_{i}n_{i}\sum_{a\mu ,bv}H_{a\mu ,bv}^{0}c_{a\mu }^{i}c_{bv}^{i}+\frac{1}{2}\sum_{a,b}\gamma_{ab}\Delta q_{a}\Delta q_{b}$$

The next approximation consists in retaining the two-center contributions only in the matrix elements. These terms are then estimated making use of the superposition of pair reference atomic densities $\rho_{0}=\rho_{0}^{a}+\rho_{0}^{b}$.

The second order expression for the KS operator is thus

(21) $$H_{a\mu ,bv}^{DFTB2}=H_{a\mu ,bv}^{0}+H_{a\mu ,bv}^{1}=H_{a\mu ,bv}^{0}+\frac{1}{2}S_{a\mu ,bv}\sum_{c\neq a,b}\Delta q_{c}\left(\gamma_{ac}+\gamma_{bc}\right)$$

Also the repulsive contribution *E* ^*rep*^ is usually taken as a sum of pair potentials

(22) $$E^{rep}=\sum_{a,b}u_{ab}^{rep}\left(\left|{\text{R}}_{a}-{\text{R}}_{b}\right|\right)$$

Finally, the last standard approximation is to consider minimal valence sets only (although auxiliary bases [53] and extended basis sets [54] have been considered also), namely *s* set for H and He, *s*, *p* set for the second and third row elements, *s*, *p*, *d* set for transition elements and *s*, *p*, *d*, *f* for rare earths.

The expansion of DFTB was carried out up to third order (DFTB3) by Elstner and co-workers [55]

(23) $$\begin{array}{l} E^{\left(3\right)}={\frac{1}{6}∭\frac{\delta^{3}E^{xc}[\rho ]}{\delta \rho (\text{r})\delta \rho \left({\text{r}}^{\prime }\right)\delta \rho \left({\text{r}}^{\prime \prime }\right)}|}_{\rho_{0}}\delta \rho \left(\text{r}\right)\delta \rho \left({\text{r}}^{\prime }\right)\delta \rho \left({\text{r}}^{\prime \prime }\right)d\text{r}d{\text{r}}^{\prime }d{\text{r}}^{\prime \prime }\\ \;={\frac{1}{6}∭\frac{\delta }{\delta \rho \left({\text{r}}^{\prime \prime }\right)}\frac{\delta^{2}E^{xc}[\rho ]}{\delta \rho (\text{r})\delta \rho \left({\text{r}}^{\prime }\right)}|}_{\rho_{0}}\delta \rho \left(\text{r}\right)\delta \rho \left({\text{r}}^{\prime }\right)\delta \rho \left({\text{r}}^{\prime \prime }\right)d\text{r}d{\text{r}}^{\prime }d{\text{r}}^{\prime \prime } \end{array}$$

Submitting the third order terms to the DFTB approximations (retaining only two body terms) yields the following expression

(24) $$\begin{array}{c} E^{\left(3\right)}=\frac{1}{6}\sum_{abc}\Delta q_{a}\Delta q_{b}\Delta q_{c}\frac{d\gamma_{ab}}{dq_{c}}=\frac{1}{6}\sum_{ab}\left(\Delta q_{a}^{2}\Delta q_{b}\Gamma_{ab}+\Delta q_{a}\Delta q_{b}^{2}\Gamma_{ba}\right)\\ \text{with}\;\Gamma_{ab}={\frac{d\gamma_{ab}}{dq_{b}}|}_{q_{a}^{0}} \end{array}$$

The matrix elements of the KS operator are

(25) $$H_{a\mu ,bv}^{DFTB3}=H_{a\mu ,bv}^{DFTB2}+S_{a\mu ,bv}\sum_{c\neq a,b}\left[\frac{1}{3}\left(\Delta q_{a}\Gamma_{ac}+\Delta q_{b}\gamma_{bc}\right)+\frac{1}{6}\Delta q_{c}\left(\Gamma_{ca}+\Gamma_{cb}\right)\right]$$

This introduces a dependence on the atomic charges *via* an integral that explicitly depends itself on the other atomic charges. Combined with a modification of the γ matrix, DFTB3 was shown [55] to provide an additional flexibility and, in particular, better proton affinities for systems involving C, H, O, N, P and other elements important for chemistry in gas phase or in solvents and, in particular, water. In contrast, DFTB3 only brings a poor improvement of the reaction barriers for proton transfer [55].

### 2.3 Parametrization issues

The parametrization of the matrix elements $H_{a\mu ,bv}^{KS}$ is achieved from DFT calculation. One starts from atomic calculations to determine the atomic KS orbitals *ϕ_aμ_* and eigenvalues *ϵ*_*aμ*_

(26) $$H_{a\mu ,a\mu }^{KS}=\epsilon_{a\mu }$$

In principle, the above atomic orbitals could provide the LCAO basis to span the DFTB Hamiltonian. These atomic orbitals are actually constrained by the addition of a confinement potential to the Kohn-Sham atomic operator under the form

(27) $$v^{con}={\left(\frac{r}{r_{0}}\right)}^{m}$$

This confinement potential may yield better transferability. The resolution of the KS equation in the presence of this potential thus defines confined atomic orbitals *ϕ̃*_*aμ*_ which will be taken as the actual DFTB/LCAO basis set.

The overlap integrals S_*aμ*,*bν*_ and the off-diagonal elements of the Hamiltonian are determined from the equivalent DFT matrix elements of the atom pairs over the above frozen atomic basis, along the inter-atomic distance *R* = |**R**_*a*_ – **R**_*b*_|

(28) $$H_{a\mu ,bv}^{0}=\langle {\tilde{\phi }}_{a\mu }\left|H_{0}^{KS}\right|{\tilde{\phi }}_{bv}\rangle \;S_{a\mu ,bv}=\langle {\tilde{\phi }}_{a\mu }|{\tilde{\phi }}_{bv}\rangle$$

The on-site second order contributions *γ*_*aa*_ are identified with the atom Hubbard parameters *U*_*a*_ and taken as the difference between the first ionization potential (IP) and the electron affinity (EA) of atom *a*

(29) $$\gamma_{aa}=U_{a}=IP\left(a\right)-EA\left(a\right)$$

The two-center integrals *γ*_*ab*_ (b≠ *a*) could in principle be calculated numerically from the exact expression provided the atomic charges and the expansion functions are known. In practice, they are expressed via an analytical damped Coulomb formula.

(30) $$\gamma_{ab}=\frac{1}{R_{ab}}-f\left(U_{a},U_{b},R_{ab}\right)$$

depending on the on-site integrals *U*_*a*_ and *U*_*b*_.

The parametrization of the repulsive term is certainly the most delicate. The initial and somewhat consistent recipe should determine this term as the difference between the purely electronic DFTB contribution to the interaction energy $\Delta E_{ab}^{DFTB(\text{elec})}$ and the total DFT interaction energy $\Delta E_{ab}^{DFT}$ of a given pair of atoms

(31) $$u_{ab}^{rep}\left(R_{ab}\right)=\Delta E_{ab}^{DFT}\left(R_{ab}\right)-\Delta E_{ab}^{DFTB\left(elec\right)}\left(R_{ab}\right)$$

Let us mention a number of attempts to improve the transferability of the parametrization beyond this basic recipe. For instance, constraints on the confinement potentials of the atomic orbitals have been used to optimize bulk electronic band spectra of all elements throughout the periodic table [56,57]. Also several authors have developed automatized algorithms [58–61] to optimize the repulsive terms in multiproperty fits to various ensembles of observables such as molecular binding energies, equilibrium geometries, bulk data band structure, elastic constants or to develop parameters dedicated to specific chemical environment [62]. Some authors also reported on-the-fly parametrization mapping the DFTB parameters on the DFT data during global optimization simulations [63]. Recently, a new scheme has been pioneered with the use of machine-learning algorithms to develop optimized parametrizations [64].

The parameters, most of the time tabulated pointwise, are finally interpolated *via* spline functions or polynomials. The main parameter sets available are the *mio* set [20], the *matsci* set [65], the *3ob* set (adapted to DFTB3) [66], the *pbc* set [67] (adapted to periodic calculations) and that of Wahiduzzaman et al. [56] for the electronic matrix elements throughout the periodic table. Note that there is a dependency between the electronic version of DFTB and the repulsive potentials. In the following, if not specified, DFTB will be used as a generic name referring either to DFTB1, DFTB2 or DFTB3.

## 3 Extensions of density-functional tight-binding

### 3.1 Non-covalent interactions

Due to its formulation in minimal basis sets and considering the present quality of the DFT functionals from which it is parametrized, DFTB tends to underestimate or even almost ignore non-covalent contributions to the energy. This includes in particular the polarization energy and the London dispersion energy. In low dimensional systems, such as 1D or 2D systems for instance, whereas the calculation of longitudinal polarizabilities can benefit of the presence of neighboring bases (mediated by the hopping integrals), the calculation of perpendicular polarizabilities may be considerably hindered due to the atomic point charge definition used in the second order term and the absence of basis sets in the orthogonal direction. In addition, the description of electrostatic fluctuations in weakly bound systems may be poorly described *via* the Mulliken charges. Improvement of electric dipole polarizabilities and polarization energies in the framework of DFTB2 [68] and DFTB3 [53,69] was proposed within the so-called Chemical Potential Equalization (CPE) scheme. The principle is based on an expansion of the energy as a response to the field in the vicinity of the field-less DFTB density

(32) $$\Delta E^{CPE}=\int {\left[\frac{\delta E}{\delta \rho (\text{r})}\right]}_{\rho_{DFTB}}\delta \rho^{CPE}\left(\text{r}\right)d\text{r}+\frac{1}{2}\iint {\left[\frac{\delta^{2}E}{\delta \rho (\text{r})\delta \left({\text{r}}^{\prime }\right)}\right]}_{\rho_{DFTB}}\delta \rho^{CPE}\left(\text{r}\right)\delta \rho^{CPE}\left({\text{r}}^{\prime }\right)d\text{r}d{\text{r}}^{\prime }$$

The response density is itself expanded over *p*-type atomic-centered Gaussian functions

(33) $$\delta \rho^{CPE}\left(\text{r}\right)=\sum_{j}d_{j}g_{j}\left(\text{r}\right)$$

Within the DFTB approximation of charge densities by discrete atomic charges, the minimization of the CPE energy is made *via* the resolution of a system of linear equations, from which the *d*_*j*_ coefficients are determined. The CPE implementation yields a modification of the Hamiltonian matrix *H*_*aμ*,*bv*_

(34) $$\Delta H_{a\mu ,bv}^{CPE}=\frac{1}{2}S_{a\mu ,bv}\left(\frac{\partial \Delta E^{CPE}}{\partial \Delta q_{a}}+\frac{\partial \Delta E^{CPE}}{\partial \Delta q_{b}}\right)$$

The DFTB3/CPE response was shown to improve intermolecular interactions involving charged and highly polarizable molecules [69].

An alternative scheme for improving polarization can be formulated in analogy with the effective core polarization operators in *ab initio* treatment. It consists in adding phenomenological atomic contributions to the DFTB energy

(35) $$E^{pol}={\sum_{a}-\frac{1}{2}\alpha_{a}\left[\sum_{b\neq a}f\left(R_{ab}\right)\frac{\Delta q_{b}{\text{R}}_{ab}}{R_{ab}^{3}}\right]}^{2}$$

This expression accounts for the polarization of atom a due to the resultant electric field created by all other atomic charges. *α*_*a*_ is the polarizability (or possibly an effective polarizability) of atom *a*, *f*(*R*_*ab*_) a cut-off function to prevent short distance divergence. *E* ^*pol*^ can be incorporated in the SCC convergence. It does not require any extra basis but may yield some overestimation of polarization contributions since the atomic polarizability correction is isotropic and may be, at least partially, superfluous (case of longitudinal polarizabilities for instance). Note however that it can be extremely helpful to properly describe MM atoms as polarizable centers in the case of combination of DFTB with MM force fields, for example in the treatment of cryogenic matrices [70].

Continuous theoretical efforts are made to derive DFT functionals describing the London dispersion [71–78]. A more phenomenological approach used in a number of applications [79–84] and systemized by Grimme et al. [85] consists in adding to the total energy specific pairadditive dispersion contributions with $1/R_{ab}^{6},1/R_{ab}^{8}\cdots$ longrange behaviour. This empirical approach was first applied for DFTB by Elsner et al. [86]. As, in standard DFTB, the dispersion energy is almost completely absent, due to the reduced basis and the functionals used for parametrization, very little double counting of the dispersion energy is expected. As for polarization, a damping cut-off is necessary to avoid attractive divergence at short distance. The form of the cut-off is strongly related to the parametrization of the repulsive potential [86–88].

As an example, the benzene dimer, unstable at the DFTB2 level, becomes stable when dispersion interactions are added [88]. Benchmarks of intermolecular interactions have been done by Christensen et al. [69] combining DFTB3, CPE and the D3 form of Grimme’s dispersion [89,90]

(36) $$E^{disp}=-\sum_{a<b}\sum_{k=3,4}s_{2k}\frac{C_{2k}^{ab}}{R_{ab}^{2k}+{\left[f_{ab}\left(R_{ab}\right)\right]}^{2k}}$$

with $C_{2k}^{ab}$ the 2k-order dispersion coefficient for the atom pair *ab*, *s*_2*k*_ a scaling factor and *f*_*ab*_ a damping function.

Finally, the energy can still be improved by modifying the Coulomb interaction. In its formulation, DFTB makes use of Mulliken definition of atomic point charges to define second and third order terms responsible for the long-range Coulomb interaction between charges fluctuations. This difference with DFT, where the Coulomb interaction is calculated from explicit 3D electronic densities, can be problematic in the case of noncovalently bonded systems, due to a delicate balance between different small contributions in the interaction energy. Among the other definitions of the atomic charges (Bader [91], Löwdin [92], …), the Class IV – Charge Model 3 (CM3) developed by the group of Truhlar [93], easy to implement within the DFTB scheme, corrects the Mulliken charges to take into account a more relevant bond polarization

(37) $$\Delta q_{a}^{CM3}=\Delta q_{a}^{Mull}+\sum_{b\neq a}^{atoms}\left[D_{ab}B_{ab}+C_{ab}B_{ab}^{2}\right]$$

where *B*_*ab*_ is the Mayer’s bond order [94] along bond *ab* and *D*_*ab*_, *C*_*ab*_ are empirical parameters. The use of CM3 charges instead of Mulliken charges, first introduced in DFTB as an *a posteriori* correction of molecular dipole values to compute IR spectra [95], was also shown to improve the long-range Coulomb interactions when used instead of Mulliken charges in DFTB equations [88]. An alternative definition of charges for DFTB was further proposed recently [96]. Let us finally mention that it was also proposed to introduce additional multipoles in the DFTB scheme to describe systems interacting with an electric field [97].

### 3.2 Spin-polarized DFTB

DFTB was initially formulated within the restricted scheme, corresponding to closed shells in which pairs of electrons *α* and *β* share the same spatial orbital. DFTB has also been formulated within spin-polarized (unrestricted DFTB) versions [98,99] with possibly different energies ∊_*iσ*_ and orbitals *φ*_*iσ*_ for different values of the spin-projection *σ*. Kohler et al. [98,99] published an atomic shell-resolved formulation. The spin-polarization (magnetization) density *m*(**r**) = *ρ*_*α*_(**r**) – *ρ*_*β*_(**r**) is discretized over the atomic centers and shell-resolved, defining atomic spin-polarization differences *m*_*al*_ = *n*_*alα*_ – *n*_*alβ*_ (*n*_*alα*_ is the electron population with *α* spin in shell *l* of atom *a*). Consistently, the charge populations *q*_*al*_ and the on-site electron-electron integrals *U*_*al*_ become shell-dependent as well as the two-center integrals *γ*_*al*,*bl*_’ which are functions of the *U*_*al*_ parameters. The spin-polarized DFTB energy (SDFTB) at second order reads

(38) $$E^{DFTB2,spin-pol}=E_{rep}+\sum_{i,\sigma }<\varphi_{i\sigma }\left|H^{0}\right|\varphi_{i\sigma }>+\frac{1}{2}\sum_{al,bl^{\prime }}\gamma_{al,bl^{\prime }}\Delta q_{al}\Delta q_{bl^{\prime }}+\frac{1}{2}\sum_{all^{\prime }}m_{al}m_{al^{\prime }}W_{all^{\prime }}$$

where the *W*_*all*_′ parameters are shell-dependent atomic constants which, similarly to the Hubbard constants, can be derived from Janak’s theorem [100].

(39) $$W_{all^{\prime }}=\frac{1}{2}\left(\frac{\partial \epsilon_{al\alpha }}{\partial n_{al^{\prime }\alpha }}-\frac{\partial \epsilon_{al\alpha }}{\partial n_{al^{\prime }\beta }}\right)$$

The above SDFTB energy corresponds to the electron spin-dependent operator

(40) $$H_{a\mu ,bv,\sigma }^{DFTB2,spin-pol}=H_{a\mu ,bv}^{0}+\frac{1}{2}S_{a\mu ,bv}\sum_{c,l^{\prime \prime }\in c}\Delta q_{cl^{\prime \prime }}\left(\gamma_{al_{\mu },cl^{\prime \prime }}+\gamma_{bl_{v}^{\prime },cl^{\prime \prime }}\right)+\delta_{\sigma }\frac{1}{2}S_{a\mu ,bv}\left(\sum_{l^{\prime }\in a}m_{al^{\prime }}W_{al_{\mu }l^{\prime }}+\sum_{l^{\prime }\in b}m_{bl^{\prime }}W_{bl_{v}l^{\prime }}\right)$$

where index *l*_*μ*_ indicates the shell associated with orbital μ on a given atom.

Note that Melix et al. [101] use a version resolved to atoms only where the spin-polarized DFTB energy is

(41) $$E^{DFTB2,spin-pol}=E_{rep}+\sum_{i,\sigma }<\varphi_{i\sigma }\left|H^{0}\right|\varphi_{i\sigma }>+\frac{1}{2}\sum_{ab}\gamma_{ab}\Delta q_{a}\Delta q_{b}+\frac{1}{2}\sum m_{a}^{2}W_{a}$$

where *W*_*a*_ is now a single atomic constant related to the population derivative of the highest occupied atomic orbital and *m*_*a*_ = *n*_*aα*_ – *n*_*aβ*_ the difference between total populations with *α* and *β* spins on atom *a*.

### 3.3 Self-interaction correction schemes

Most of standard DFT functionals undergo self-interaction error (SIE) which stems from the fact that the self-exchange contributions in the functionals do not cancel the self-Coulomb contribution. In its original formulation, DFTB meets the same problem. The SIE is responsible for several major errors of standard DFT (and LDA in particular), namely (i) the deviation of the asymptotic potential from $-\frac{1}{r}$ which induces electron overdelocalization, (ii) an underestimation of the HOMO-LUMO gap and (iii) the non linearity and derivative continuity of the energy dependence of the system upon the number of electrons [102].

Several schemes have been proposed to cure the SIE of standard DFT, involving full self-interaction corrections [103], the GW formalism [104], or using hybrid functionals including a part of Hartree-Fock exchange [105]. Other schemes to correct LDA calculations consist in adding corrections Δ*E* ^*SIC*^ calculated within the Hubbard model and on-site electronelectron effective interactions *U*_*a*_. This has yielded the LDA+U schemes which have also been declined using *l*-resolved electron-electron screened interactions *U*_*al*_ – *J*_*al*_ [106]. An alternative so-called pseudo-SIC scheme [107–109] consists in expressing the corrections via the projections of the KS orbitals onto atomic states concerned with the highly correlated shells (*d* and/or *f* electrons). Houharine et al. [110] transposed those LDA+U and pseudo-SIC corrections within the spin-polarized DFTB formalism. For example, the pseudo-SIC correction reads

(42) $$\Delta E^{pSIC}=-\alpha \sum_{a}\sum_{l\in a}\frac{\left(U_{al}-J_{al}\right)}{2}\sum_{\sigma }\sum_{a\mu ,av\in l}{\left(n_{a\mu ,av}^{\sigma }\right)}^{2}$$

where *U*_*al*_ – *J*_*al*_ is taken from atomic DFT calculations and $n_{a\mu ,b\nu }^{\sigma }$ is a matrix generalization of the basis functions Mulliken atomic occupation numbers for a given shell *l* and a given spin projection *σ*. *α* is here an empirical scaling parameter. Analogous expressions were given for the LDA+U schemes either in the fully localized (FLL) or in the mean-field (AMF) limits. All these corrections rely on the fact that the largest contribution to the SIE is that corresponding to electrons in localized shells. Those contributions to the energy may bring significant improvement. For instance they allow for a gap opening in the strongly correlated antiferromagnetic phase II of bulk NiO, even though the gap remains underestimated. Conversey the corrected magnetic moments show magnitudes comparable with the experimental ones. Further corrections, based on the trace of the idempotent expression *ρ̂* – *ρ̂* *S* *ρ̂* were proposed to tackle the derivative continuities of the energy as a function of the electron number. Test calculations over several aromatic molecules with CuS substitutive contacts show that such corrections strongly increase the HOMO-LUMO gap which becomes quite consistent with its thermodynamic charge definition *E*(*N* + 1) − 2*E*(*N*) + *E*(*N* − 1).

Another extension of DFTB in relationship with the SIE problem concerns specific classes of systems such as cationic molecular clusters which consist of well identified subsystems. In such cases, delocalization can be strongly overestimated in DFTB as in standard DFT. The single electron picture may also present incorrect dissociation and, since it equally distributes the charge on the separated subsystems (case of two identical subsystems), it may induce spurious Coulomb repulsion at intermediate and long distance separation [10]. Those drawbacks can be circumvented when combining DFTB with Configuration Interaction within a valence bond framework, namely describing the global system *via* a multiconfigurational wavefunction expanded on charge-localized configurations:

(43) $$\Psi_{0}^{+}=\sum_{A}C_{A}a_{A}^{HOMO}\Psi_{0}=\sum_{A}C_{A}\Psi_{A}^{+}$$

where Ψ_0_ is the wavefunction of the neutral cluster and $a_{A}^{HOMO}$ the electron annihilation operator of the HOMO on fragment *A*. The CI problem is then restricted to a secular equation in the charge localized basis

(44) $$\sum_{B}\left(H_{AB}^{CI}-E_{0}S_{AB}^{CI}\right)C_{B}=0$$

where *H* ^*CI*^ and *S* ^*CI*^ are the Hamiltonian and overlap matrices respectively in the charge-localized configurations basis $\left\{\Psi_{\text{A}}^{+}\right\}$. The dimension of the CI matrix is only the number of fragments. In this approach, the diagonal terms of the Hamiltonian represent the energies of fragment-localized charge configurations, while the non dynamical correlation arising from the charge resonance and determining the extension of charge fluctuation is mediated by the hopping integrals in the CI resolution. Note that this valence bond CI formulation is well suited to investigate hole transfer through extended system since it provides a naturally quasi-diabatic framework where the hole dynamics is promoted by the hopping integrals [111,112].

A similar partitioning scheme was the principle of the DFTB coarsegrained based approach developed by Elstner et al. [113–116] to study charge transfer in DNA. In this approach, the MOs are calculated independently for each fragment (the fragment orbital approach [117,118]). The diagonal elements are estimated from DFTB2 single particle energies and the hopping term between two fragments is calculated as

(45) $$H_{AB}^{CI}=\;<\varphi_{HOMO}^{A}\left|H^{0}\right|\varphi_{HOMO}^{B}>$$

where $\varphi_{HOMO}^{A}$ is the HOMO of the charged fragment *A* in configuration $\Psi_{\text{A}}^{+}$ and *H* ^0^ the DFTB1 Kohn-Sham Hamiltonian. The charge mobility in DNA could be described by non-adiabatic MD in a mean field approach with a refined version of this coarse grain model [119].

An alternative scheme in a similar philosophy is that adapted from the constrained-DFT scheme [120–122], in which the orbitals of the chargelocalized configurations $\Psi_{\text{A}}^{+}$ are calculated variationally within the DFTB scheme, minimizing a Lagrangian with respect to the orbitals $\varphi_{i}^{A}$ with constraint of charge localization on a given fragment *A*

(46) $${ℒ}^{A}=E\left(\left\{\varphi_{i}^{A}\right\}\right)-\sum_{ij}\epsilon_{ij}^{A}\left(<\varphi_{i}^{A}|\varphi_{j}^{A}>-\delta_{ij}\right)+\lambda^{A}\left(\sum_{i}<\varphi_{i}^{A}\left|P^{A}\right|\varphi_{j}^{A}>-N_{A}\right)$$

where $E\left(\left\{\varphi_{i}^{A}\right\}\right)$ is the DFT energy and the second term ensures the MO orthonormality constraint. The last term is the expression of the charge localization constraint, with *λ* ^*A*^ a Lagrange parameter, *P* ^*A*^ a projector of the density on the fragment carrying the charge and *N*_*A*_ the number of electrons fixing the charge localization on fragment *A*. Following Wu and Van Voohris [123,124] the hopping integrals can be computed from the different charge-localized MO coefficients and the Lagrange constraints parameters. The combination of this approach within the DFTB approximations gives the DFTB-CI method [125,126]. This approach differs from the previous coarse-grained one [113] in the sense that each charge localized configuration is calculated self-consistently, thus including relaxation and polarization of the neutral fragments by the charged one. From the computational point of view, the Lagrangian optimization has to be repeated for each fragment, which is more time-consuming than simple DFTB.

### 3.4 Long-range corrected DFTB

The long-range corrected DFT scheme (LC-DFT) has also been quite fruitful in curing DFT deficiencies. It is based on a range separation of the electron-electron Coulomb interaction. The short-range part is treated *via* a DFT exchange-correlation functional while the long-range contribution can receive a better treatment, for instance *via* exact Hartree Fock exchange, contributing to cancellation of the SIE. LC-DFT achievements are obviously more general since they also address issues of long-range correlation either via a higher level correlation functional or even via combinations with Wavefunction type calculations [4,14–16] in order to deal with the dynamical and non-dynamical contributions to electronic correlation. The longrange corrected DFTB scheme (LC-DFTB) was formulated by Lutsker et al. [127] using a Yukawa long-range/short-range type separation of the Coulomb operator

(47) $$\frac{1}{r_{12}}=\frac{\exp\left(-\omega r_{12}\right)}{r_{12}}+\frac{1-\exp\left(-\omega r_{12}\right)}{r_{12}}$$

This scheme depends on a separation range parameter *ω*. Using the specific DFTB approximations, the Hamiltonian can be cast as

(48) $$H_{a\mu ,bv}=H_{a\mu ,bv}^{0}+\frac{1}{4}\sum_{c\lambda ,d\tau }\rho_{c\lambda ,d\tau }S_{a\mu ,bv}S_{c\lambda ,d\tau }\left(\gamma_{ac}+\gamma_{ad}+\gamma_{bc}+\gamma_{bd}\right)$$

(49) $$-\frac{1}{8}\sum_{c\lambda ,d\tau }\rho_{c\lambda ,d\tau }S_{a\mu ,c\lambda }S_{d\tau ,bv}\left(\gamma_{ab}^{lr}+\gamma_{cd}^{lr}+\gamma_{ad}^{lr}+\gamma_{db}^{lr}\right)$$

where *γ_ab_* is the two center second order integral calculated with the full Coulomb potential while $\gamma_{ab}^{lr}$ is calculated with the long-range part only.

Lutsker et al. [127] benchmarked applications with DFTB parameters extracted from LC-DFT calculations involving the LDA exchange functional and the local PBE form of correlation for a set of organic molecules. They showed that, similarly to LC-DFT schemes, LC-DFTB largely cures the delocalization problem attributed to SIE. As a consequence, a number of properties of the systems are significantly improved, such as the energy of the frontier orbitals, and consequently the estimations of the ionization potentials based on the HOMO energies, the HOMO-LUMO gap, or electrical properties (longitudinal polarizabilities of polyacenes). The LCDFTB also significantly improves the density of states with respect to photoelectron spectroscopy data. The ordering of the orbitals in delicate cases can still turn out to be incorrect and electron affinities still in default, either due to inherent DFTB approximations (minimal basis set, retain of two-center integrals only) or to the PBE-based parametrization. The improvement of excited electronic states with the LC-DFTB correction is discussed in section 3.6.

### 3.5 DFTB in hybrid and QM-MM methods

DFTB has also been involved in schemes were the most active atoms/molecules are treated *via* a higher level quantum-mechanical (QM) scheme while the largest part of the system (large molecule or solvent) is treated at a lower level of approximation, generally *via* molecular mechanics (MM) potentials or force fields (FF). It should be noted that DFTB, involving two-center approximations, atom-based charges and two-atom repulsive interactions, is very well suited for combination with force fields. The inclusion of point charges in force field is quite straightforward since DFTB is itself based on point charges for the QM atoms. Thus there have been adaptations of DFTB (QM method) within various MM packages such as CHARMM [128], AMBER [129] or GROMACS [130].

Another type of QM-MM combination was adapted to investigate the dynamics of molecules or clusters in a cryogenic environment, namely rare gas inert matrices. This scheme relies on the definition of (possibly) anisotropic two-body interactions between the active atoms and the rare gas atoms added to the DFTB-KS operator in the AO basis, the description of inert atoms interaction (Rg-Rg) *via* a pair potential, and the inclusion of the polarization response of the Rg atoms. Inclusion of the latter can be handled *via* atomic polarization operators (see Equation 35) which can be finalized adding the following contributions to the initial electrostatic/exchange correlation contributions to the DFTB2 *γ* matrix

(50) $$\gamma_{ab}^{pol}=-\sum_{c\in Rg}\alpha_{c}f_{ac}\left(R_{ac}\right)f_{bc}\left(R_{bc}\right)\frac{{\text{R}}_{ab}{\text{R}}_{bc}}{R_{ab}^{6}R_{bc}^{6}}$$

where the rare gas atomic polarization *α*_*c*_ and the cut-off functions *f*_*ac*_(*R*) between active atoms and Rg inert atoms are introduced [70]. Such scheme proved able to describe the influence of the matrix on the structures of molecular complexes such as water clusters in interaction with polycyclic aromatic hydrocarbons [131]. Another combination has also been explored combining DFTB as the low level description with DFT as the high level method [132].

Finally, let us mention that environmental effects can also be taken into account through a polarizable continuum model (for both ground and excited states) [133].

### 3.6 Excited states and time-dependent DFTB

In the framework of Density Functional Theory, the access to excited states is given by the electronic response, based on the time-dependent Kohn-Sham equation

(51) $$i\frac{\partial \varphi_{j}\left(\text{r},t\right)}{\partial t}=\left(-\frac{1}{2}\Delta +v_{KS}\left[\rho \left(\text{r},t\right)\right]\right)\varphi_{j}\left(\text{r},t\right)$$

The linear response TD-DFTB was originally developed by Niehaus et al. [134] as a DFTB analogue of the linear response TD-DFT [135,136]. Excitation energies are given as the eigenvalues Ω_*J*_ of the following matrix equation

(52) $$\left(\begin{array}{cc} \text{A} & \text{B}\\ \text{B} & \text{A} \end{array}\right)\left(\begin{array}{l} \text{X}\\ \text{Y} \end{array}\right)=\Omega_{J}\left(\begin{array}{cc} \text{I} & 0\\ 0 & -\text{I} \end{array}\right)\left(\begin{array}{l} \text{X}\\ \text{Y} \end{array}\right)$$

where **I** is the identity matrix, **A** and **B** are matrices with the following elements

(53) $$A_{ik,jl}=\left(\epsilon_{j}-\epsilon_{k}\right)\delta_{ij}\delta_{kl}+2K_{ik,jl}$$

(54) $$B_{ik,jl}=2K_{ik,jl}$$

where indices *i*, *j* and *k*, *l* label occupied and virtual orbitals respectively, with energies *ϵ*_*i*_, *ϵ*_*j*_ and *ϵ*_*k*_, *ϵ*_*l*_. The coupling matrices **K**, depending on the spin configuration, are determined within the DFTB scheme [134] using the Mulliken approximation to compute transition dipoles. The first application of the linear response TD-DFTB was reported in ref [134]. Absorption spectra were computed for neutral polyacenes ranging in size from naphthalene to heptacene and compared with experimental as well as TD-DFT data. Vibrationally resolved UV/Vis spectra of various aromatic and polar molecules were calculated using TD-DFTB excitation energies and analytical gradients in ref [137]. The results of TD-DFTB were found in a very good agreement with the TD-DFT calculations using local functionals.

Several extensions were developed in the framework of the linear response TD-DFTB. Spin-unrestricted TD-DFTB [138,139] has been implemented in order to study absorption spectra of open-shell systems. Conventional TD-DFTB fails to properly describe PES for charge transfer states. TD-DFTB was combined with LC-DFTB [140–142] to benefit from the range separation improvement for excited states that, in particular, leads to the recovering of a correct -1/*r* behaviour of the potential. Also, incorporation of intra-atomic exchange integrals [139,143] was shown to improve the transitions energies both towards triplet and singlet TDDFTB states. Calculation of spin-orbit coupling was interfaced by Gao et al. [144] for TD-DFT approaches, including TD-DFTB. From a computational efficiency point of view, intensity-selected TD-DFTB has been introduced by Rüger et al. [145], delivering similar accuracy as the linear response TD-DFTB, but at a lower computational cost. More details about the TD-DFTB method as well as some other examples of applications can be found in the review paper of T. A. Niehaus [146].

Further improvements were done in order to derive intermolecular excitonic transfer couplings according to the Förster mechanism, implying a formulation of the interaction integral between the transition dipoles of the interacting molecules *A* and *B*

(55) $$J_{AB}^{m}=\iint \frac{<\Psi_{A}^{0}|\hat{\rho }(\text{r})|\Psi_{A}^{m}><\Psi_{B}^{m}\left|\hat{\rho }\left({\text{r}}^{\prime }\right)\right|\Psi_{B}^{0}>}{\left|\text{r}-{\text{r}}^{\prime }\right|}d\text{r}d{\text{r}}^{\prime }$$

where $\Psi_{A}^{m}$ is the intramolecular excited state on *A* correlated with the exciton band. Within the DFTB formalism this integral becomes [147,148]

(56) $$J_{AB}^{m}=\sum_{a\in A}\sum_{b\in B}Q_{a}^{m}\gamma_{ab}Q_{b}^{m}$$

where quantities $Q_{a}^{m}$ are atomic many-body transition charges determined within the TD-DFTB scheme.

Another extension has also been opened for charged molecular clusters in the framework of the DFTB-CI scheme (see above). Initially developed to investigate the ground state, it also delivers excited states as higher roots of the CI matrix. The formalism has been extended in order to provide a better description of the ionic excited states considering in the basis of charged localized configurations, not only the removal of an electron from the HOMO of the charged fragment, but also electron removal from sub-HOMO occupied orbitals $\varphi_{i}^{A}$, yielding a more general wavefunction [149]

(57) $$\Psi_{0}^{+}=\sum_{A,i\in occ}c_{Ai}a_{Ai}^{†}a_{HOMO}\Psi_{A}^{+}=\sum_{A,i\in occ}c_{Ai}\Psi_{Ai}^{+}$$

This improvement *vs* the simple initial scheme restricted to the HOMO orbital becomes important for clusters or stacks of large molecules, presenting a small orbital separation below the HOMO. Moreover, it allows to incorporate not only the excited states of the charge transfer band, but also those correlated with local excitations on the fragment ions, and their coupling. This scheme has been applied to ionic clusters of polyaromatic hydrocarbon molecules and shown to yield satisfactory excited states potential energy surface in the full geometry range up to intermolecular dissociation [149].

### 3.7 Global exploration of the energy landscape and dynamics

Global exploration of the potential energy surface (PES) or energy landscape is now standard either using Monte Carlo (MC) or molecular dynamics (MD) evolution schemes. While MC only requires the knowledge of the total DFTB energy, the energy gradient is needed in MD. In the widely used DFTB2 approximation, the expression of the gradient is

(58) $${\text{F}}_{a}=-\sum_{b}\frac{\partial u_{ab}^{rep}}{\partial {\text{R}}_{a}}-\sum_{i}\sum_{a\mu ,bv}n_{i}c_{a\mu }^{i}c_{bv}^{i}\left(\frac{\partial H_{a\mu ,bv}^{0}}{\partial {\text{R}}_{a}}-\left(\epsilon_{i}-\frac{H_{a\mu ,bv}^{1}}{S_{a\mu ,bv}}\right)\frac{\partial S_{a\mu ,bv}}{\partial {\text{R}}_{a}}\right)-\Delta q_{a}\sum_{b}\frac{\gamma_{ab}}{\partial {\text{R}}_{a}}\Delta q_{b}$$

Note that ground state PES gradients are also available in various extended versions of DFTB such as DFTB3 [55], spin-polarized DFTB [99], CI-DFTB [150] or when LDA+U or pSIC-corrections are included [110].

In large systems like extended and/or flexible molecules, atomic or molecular clusters, structural intuition is delicate, due to the large number of degrees of freedom. Finding the most stable structure (global minimum) and possibly secondary metastable minima might become a challenging task [151] and requires global optimization (GO) schemes with no *a priori* knowledge of the final structure. A variety of them have been coupled with DFTB and often require the computation of millions of single point energies and possibly gradients for various geometries. A first family of GO schemes rely on genetic algorithms [152] often used to search for atomic cluster structures [63,153–157]. Simulated annealing [158] as well as basinhopping schemes [159,160] have also often been used either in their standard form [161–163] or improved versions like the modified basin hopping [164,165] or the Tsinghua global minimum algorithms [166]. Other approaches rely on the exploration of the complex potential energy surface (PES) with either MC or MD simulations, which are combined with regular local optimization of the visited geometries as done for ammonium/water clusters [167]. Reaching the bottom of the lowest energy PES basin requires low temperature exploration, but, in such case, the system might be trapped in local minima with vanishing possibility to overcome barriers. An alternative consists in running several simulations at different temperatures [168] and to allow for replica exchange (RE) between the latter following a Boltzmann criterion leading to Parallel Tempering (PT) schemes for MC [169] or MD [170,171]. In the context of DFTB, Parallel-Tempering schemes have appeared quite powerful in finding local minima for atomic and molecular clusters [172–175].

Obviously, MD is also be used to follow the dynamical aspects of the system, for instance to simulate a reaction, collision and/or fragmentation (see section 4.7). A Car-Parrinello version of DFTB molecular dynamics was also implemented [176] as well as biased dynamics schemes like metadynamics [177–179]. Thermodynamical quantities can also be calculated. For instance, DFTB has been combined with the multiple histogram method of Labastie and Whetten [180] to derive the entropy and the heat capacity curves of finite clusters and complexes [181].

IR spectra can be determined in the harmonic approximation, calculating the eigenmodes of the mass-weighted Hessian matrix. However, MD allows to go beyond the harmonic approximation, integrating the IR absorption spectra at finite temperature on-the-fly via the Fourier transform of the autocorrelation function of the electric dipole ***μ*** along the trajectories [182]

(59) $$I\left(\omega \right)\;\propto \;\omega^{2}\int_{-\infty }^{+\infty }\text{d}t\langle \mu \left(0\right)\cdot \mu \left(t\right)\rangle \;e^{i\omega t}$$

where < > indicates a statistical average to minimize spurious correlations. Let us mention that anharmonic effect can also be obtained from *a posteriori* treatment of cubic and quartic derivatives of the PES [183,184]. However, the quartic constant can only be obtained at the DFT level for small systems, whereas their computation at the DFTB level could allow for the application of such approaches to larger molecules [185,186].

Finally, recent advances concern the dynamics of excited states. In order to propagate the classical trajectory on a given excited PES, the TD-DFTB excited states energy gradients were developed. The derivation relies on the so-called Z-vector method, which was initially introduced by Furche and Ahlrichs [187,188] to compute analytical forces for the TD-DFT excited states. The procedure was further used to derive TD-DFTB gradients by Heringer et al. [189,190] and led to the final expression published in ref [137].

Non-Adiabatic Molecular Dynamics (NAMD) coupling electronic and nuclear motions has been implemented in the framework of mixed approaches within a DFTB/TD-DFTB quantum description of the electrons and classical nuclei.

Mostly two directions have been followed. In the first approach, the electronic motion is described by the explicit propagation of electronic wavepackets mediated by the time-dependent Schrödinger equation (or equivalent schemes) while the nuclei are propagated in a mean timedependent electronic potential *E*[*ρ*(**R**, **r**, *t*] (Ehrenfest-like propagation of nuclei).

(60) $$M_{a}\frac{d^{2}{\text{R}}_{\text{a}}}{dt^{2}}=-\nabla_{a}E\left[\rho \left(\text{R}\left(\text{t}\right),\text{r},t\right)\right]$$

where *ρ*(**R**(**t**),**r**,*t*) is now the time-dependent electronic density corresponding to molecular orbitals *φ*_*i*_(**R**(**t**),**r**,*t*) obeying the time-dependent DFTB equation. A version of mean potential non-adiabatic dynamics with DFTB was first derived by Niehaus et al. via a variational treatment of the equation of motion (EOM) and the definition of a Lagrangian from which the time-dependant equations can be derived [191]. Mean potential NAMD schemes can also be derived based on the Liouville-von Neumann equation

(61) $$i\frac{\partial \hat{\rho }\left(\text{R}\left(\text{t}\right),\text{r},t\right)}{\partial t}=\left[H_{KS},\hat{\rho }\left(\text{R}\left(\text{t}\right),\text{r},t\right)\right]$$

One may cite the NAMD scheme derived by Jakowski [192] and other developments made in the context of electronic transport [193,194].

The second approach relies on the Tully’s Trajectory Surface Hopping (TSH) scheme [195,196]. Here, the motion is propagated on the adiabatic PES of the TD-DFTB excited states, with probabilities to hop between states Ψ_*m*_ and Ψ_*n*_ determined by the non-adiabatic couplings

(62) $$<\Psi_{m}\left|\frac{\partial }{\partial Q}\right|\Psi_{n}>$$

along some relevant coordinate *Q* (possibly a generalized coordinate along the trajectory).

The first article describing methodological as well as development aspects of TSH (in the fewest-switches or FSSH version) coupled with TD-DFTB electronic structure calculation was published by Mitrić et al. [197]. DFTB, as a density functional method, is not initially designed to use wavefunctions to compute properties. Nevertheless, the most common practice is to use the excited state wavefunctions associated with the single excitation configuration interaction (CIS) approximation spanning the TD-DFTB excited states to determine the non-adiabatic couplings presented above [197–201]. This can be achieved through the calculation of the overlap of the CIS electronic wavefunctions between nuclear time steps *t* and *t* Δ*t*. This procedure is described within the framework of TD-DFTB by Humeniuk and Mitric [200]. Several implementations of FSSH are available within various open-source DFTB codes, such as DFTBaby [200], DFTB+ coupled with the NewtonX or PYXAID packages [201,202] and DeMonNano [203].

## 4 Applications

### 4.1 Small molecules

Small and medium size molecules can be treated safely *via* DFT or wavefunction methods. Nevertheless, determination of their ground state properties (structure, energetics, dipole moments, binding energies, vibrational spectra, proton affinities, hydrogen bonds, proton transfer barriers) provides benchmarks for checking the accuracy of DFTB *vs* DFT, wavefunction calculations (MP2, MP4, Coupled-Cluster or multi-reference CI) or experimental data. Moreover, generic small molecules are often building blocks of larger and/or new systems for which one may expect some transferability. Finally, since reference data are available they also allow to evaluate the various DFTB improvements including the parametrization issues.

In the early DFTB2 versions, the average performances for a set of small organic molecules [204] were found to be 0.017 Å for bond lengths, 2 degrees for bond angles, 5 kcal/mol for dissociation energies and relative errors in the range 6–7 percent on harmonic vibrational frequencies. Recent studies focused on the barrier heights and energetics of reactions with organic molecules [205,206]. The description of the isomers (epimers) of glucose at the DFTB level has also been compared with DFT and wavefunction results: the agreement between structural parameters was shown to be good except when hydrogen bonds are present [207]. The goal was to study large carbohydrate networks which would be out of reach with DFT approaches. Very systematic benchmarks were produced recently to assess the accuracy of the DFTB3 and LC-DFTB2 methods [24,208] covering reference molecule sets. So far, the DFTB3 level appears as the DFTB reference, including benchmarks of proton affinities and hydrogen bonding in organic and biological molecules [209]. Systematic benchmarks of DFTB3 (with the corresponding OB3 parametrization and possibly completed by the addition of the D3 dispersion), LC-DFTB2 with re-optimized parametrization (named OB2^0.3^) and DFTB2 (with the *mio* parameters set), have been recently performed in particular for about 70 neutral closed shells molecules containing C, H, N, and O including the G2/97 set [210]. Structural reference data originate from DFTB-B3LYP/6-31+G(d,p) calculations while the G3B3 data [211] are the reference for energetics. All DFTB methods perform quite well for geometries. The mean absolute deviations (MAD) of bond lenghts *vs* B3LYP calculations are around 0.01 Å with DFTB2, and around 0.005 Å with DFTB3 and LC-DFTB2, deviations for bond angles are in the range 0.6–0.7 degrees for all methods, while deviations for dihedral angles are within 2–3 degrees. Atomization energies have been compared with the reference data G3B3/MP2 [211]. A net improvement is observed for DFTB3 and LC-DFTB2 methods with a mean deviation of 5–6 kcal/mol *vs* 20 kcal/mol for the initial DFTB2 scheme. Conversely, all DFTB methods provide deviations of reaction energies in the range 8–10 kcal/ mol. Frequencies of selected stretch vibrations show a much better accuracy with DFTB3 and LC-DFTB2 methods, with MADs of 35 and 42 cm^−1^ respectively, than with DFTB2 (MAD of 156 cm^−1^). Comparisons against the experimental molecular data of the Jorgensen set [212] for geometries and energetics show deviations with the same order of magnitude as above, while the dipole moments deviations are in the range 0.3–0.4 Debye, whatever the DFTB level.

Other benchmarks have been done for molecule subsets (closed shells including C, H, O and N atoms only) of the GMTKN0 database [213–216] dedicated to main group thermochemistry and non-covalent interactions of small molecules and even proteins. Errors on a set of reaction energies obtained with DFTB2 and LC-DFTB2 are in the range 0.5–14 kcal/mol, while DFTB3 performs slightly better. Hydrogen binding energies show mean deviations of 3.5 kcal/mol with DFTB3 and 5–6 kcal for LC-DFTB2. Deviations for proton affinities of acidic oxygen (nitrogen) species are 3.7 (17.4) kcal/mol with DFTB2, 3.7 kcal/mol (6.9 kcal/mol and 2.9 kcal/mol, respectively, with modified NH parameters) for DFTB3 and around 8.5 kcal/mol with LC-DFTB2, while proton transfer barriers are in the range 2–3 kcal/mol with DFTB2 and LC-DFTB2 instead of 1 kcal/mol for DFTB3. Finally non-covalent interactions in molecular complexes corresponding to the S66 set [217] were benchmarked against the CCSDT/CBS limit, showing a deviation of 0.82 kcal/mol and around 2.3 kcal/mol for LC-DFTB2 with dispersion.

Other families of molecules outside the above sets have been investigated. Geometries and relative energies were determined for organometallic complexes, the electronic structure of which may be delicate to describe [218–221]. Investigating a series of organometallic complexes with SDFTB2, Zheng et al. [220] estimated an average accuracy of 0.1 Å for bond lengths, 10 degrees for bond angles, finding significant average errors on dissociation energies (25–50 kcal/mol) and on transition energies between spin isomers (10–40 kcal/mol). More recently, it was shown on the example of zinc and manganese complexes [219], that the DFTB3 level (here with *l*-dependent Hubbard integrals) strongly reduces the mean errors down to 0.03 Å for the bond lengths and 2–5 kcal/mol for the energetics, referencing to B3LYP and even G3B3/MP2 data, the largest errors corresponding to interactions of the metal ions with highly charged or polarizable ligands.

One can also cite the specific case of halogens. Kubar et al. [222] benchmarked SDFTB2 parametrization against the experimental CCCBDB database [223] for a series of halogen-containing organic molecules and found absolute errors of 0.045 Å for bond lengths, below 3.6 degrees for bond angles, and 26 and 16 cm^−1^ for stretching and bending modes respectively. Conversely, reaction energies could present significant errors, in the range 3–30 kcal/mol depending on the type of rearrangement. Kubillus et al. [224] benchmarked DFTB3+D3(X) results against the specific *X*40 halogen database of Rezac et al. [225] and showed that, depending on the halogen atom, DFTB3+D3 could provide mean absolute errors smaller than 0.035 Å and 3 degrees for bond lengths and bond angles respectively, and 25–45 cm^−1^ for vibrational frequencies with a larger error (≈ 108 cm^−1^) for bromine. Atomization energy errors were found in the range 5–17 kcal/mol, significantly large, however somewhat better than PBE/def2-sv results for Cl and F.

The performance of DFTB regarding the computation of ionization potentials and electron affinities has also been evaluated. Darghouth et al. achieved DFTB3 calculations on a set of small and medium size organic molecules with potential photovoltaic interest [226], comparing with experimental data. Determination of total energy differences (Δ*SCF*) gave deviations within ± 0.75 eV and ± 0.49 eV for IPs and EAs respectively, while somewhat better results were obtained when using Koopmans’s theorem, namely IPs and EAs errors within ± 0.45 eV and ± 0.33 eV, respectively.

**Figure 2:**
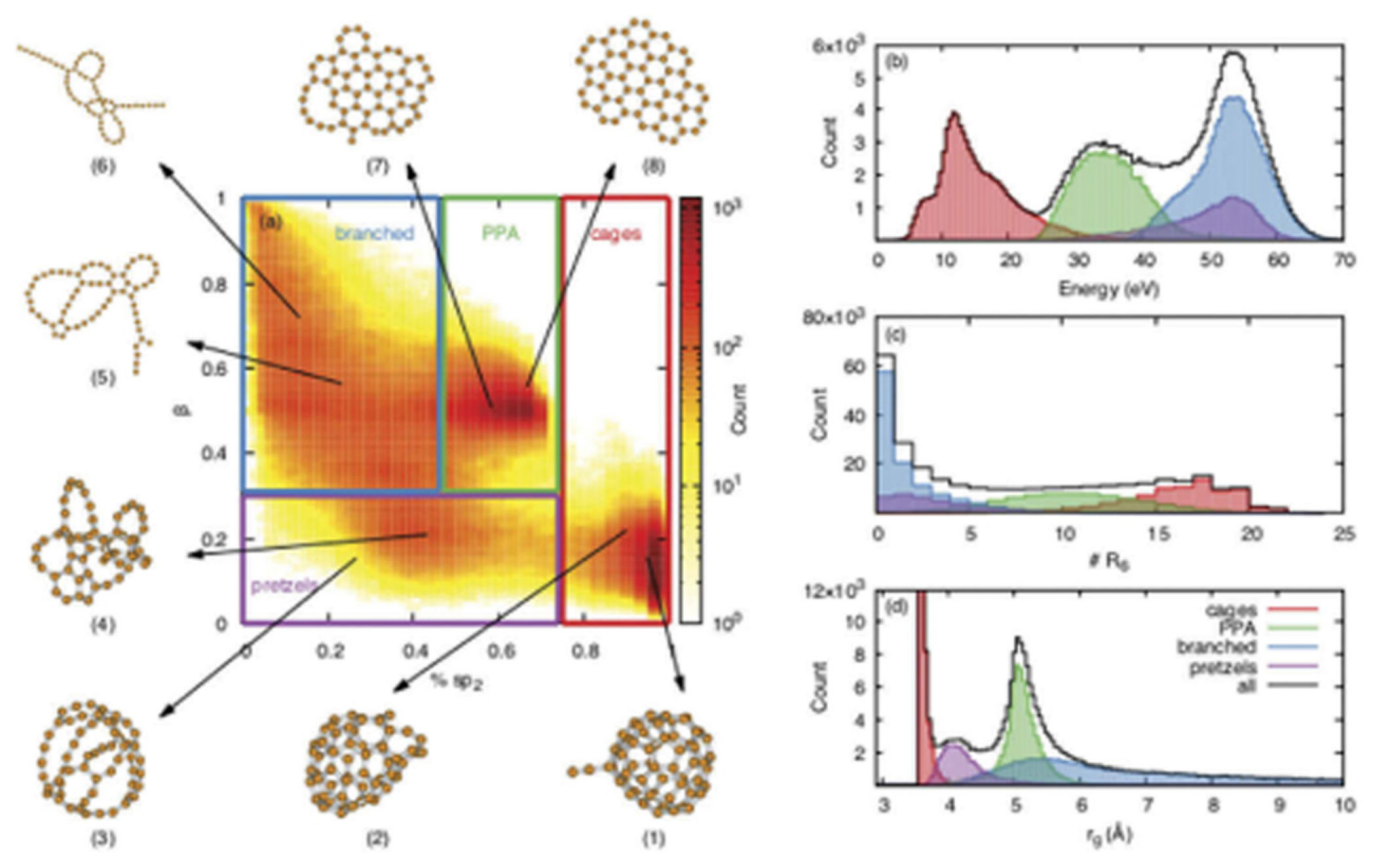
Distributions of the samples of C_60_ isomers based on specific order parameters. Left: twodimensional distribution as a function of the *sp*2 hybridization fraction and asphericity parameter *β*. The boxes classify the four structural families, cage, planar polycyclic aromatic (PPA), pretzel, and branched. Right: one-dimensional distributions as a function of isomer energy (top), number of 6-member cycles (medium), and gyration radius (bottom). Reproduced from [232].

Let us finally mention the case of pure individual carbon clusters, for which the electronic structure, the relative energies and vibrational spectra have been investigated [227–230]. Such systems have sustained a lot of interest due to their relevance in the astrophysical, atmospherical and nanomaterial domains. One can cite for instance the important case of buckminsterfullerene C_60_ which has been detected in space. The interest of DFTB for small and medium size molecules is that its efficiency allows the description of large populations of isomers. For instance, an automatic search of benzene isomers has led to the identification of 7000 isomers and 26229 transition structures [231]. DFTB was also used to explore hundreds of thousands of carbon clusters isomers containing 24 to 60 carbon atoms, allowing a classification into structural families and a statistical characterization of their spectroscopic properties (see Figure 2) [232].

### 4.2 Large molecules and biomolecules

One of the main goals behind the development of DFTB was the possibility of modeling systems much larger than those accessible in DFT, while maintaining an electronic scale description of the systems studied. In this framework, many studies have focused on the modeling of nucleic acids and proteins [86,233]. In the case of nucleic acids, most DFTB studies are concerned with the interaction of DNA fragments with different systems. Examples include investigations of the interaction between small DNA fragments and anticancer drugs [234–236], and also between a DNA basis and a carbon nanotube [237]. Charge transport through a short DNA oligomer has also been investigated [238]. It should be noted that some authors have reported that the DFTB2+D method fails to adequately describe deoxyribose and ribose sugar ring pucker [239,240]. In the case of enzymes, studies involving DFTB mainly concern reaction mechanisms carried out using the QM/MM method, with DFTB making it possible to include in the QM reactive zone much more reactive groups than the DFT/MM calculations [24]. The implementation of the DFTB method in codes widely used in hybrid DFT/MM calculations has considerably facilitated access to this method for such hybrid studies. Very different enzymatic mechanisms have been explored, such as proton transfer reactions or proton storage [241,242], histone methylation [243], C-terminal residue cleavage [244], amide hydrolysis [245], glycosylation/deglycosylation [246,247], inactivation of a new tuberculosis target [248], hydrolysis of organophosphorus [51] or proton-coupled electron transfer reactions [249]. One can also cite DFTB studies aimed at investigating substrate promiscuity [250], ion binding and transport by membrane proteins [251], proton distribution over multiple binding sites of a membrane protein [252] or evaluating the pKa of protein residues [253]. The efficiency of the DFTB/MM method also allows the comparison of catalytic pathways [254,255] and the contribution to protein design [256]. Note that it has been reported that, although the DFTB2 method is accurate with regard to protein structure, it sometimes differs from more precise calculations with regard to the electronic states on which it converges [132]. Even the DFTB3 level does not allow a good evaluation of vertical transition energies in the case of the Red Fluorescent protein [257]. Some studies focus on other biologically relevant systems, such as drug [258] or plasma species [259]. To further reduce the computational cost of such biochemical processes studies, different research groups are working at coupling DFTB with linear scaling methods, such as the fragment molecular orbital (FMO) one [260,261].

### 4.3 Clusters and nanoparticles

**Figure 3:**
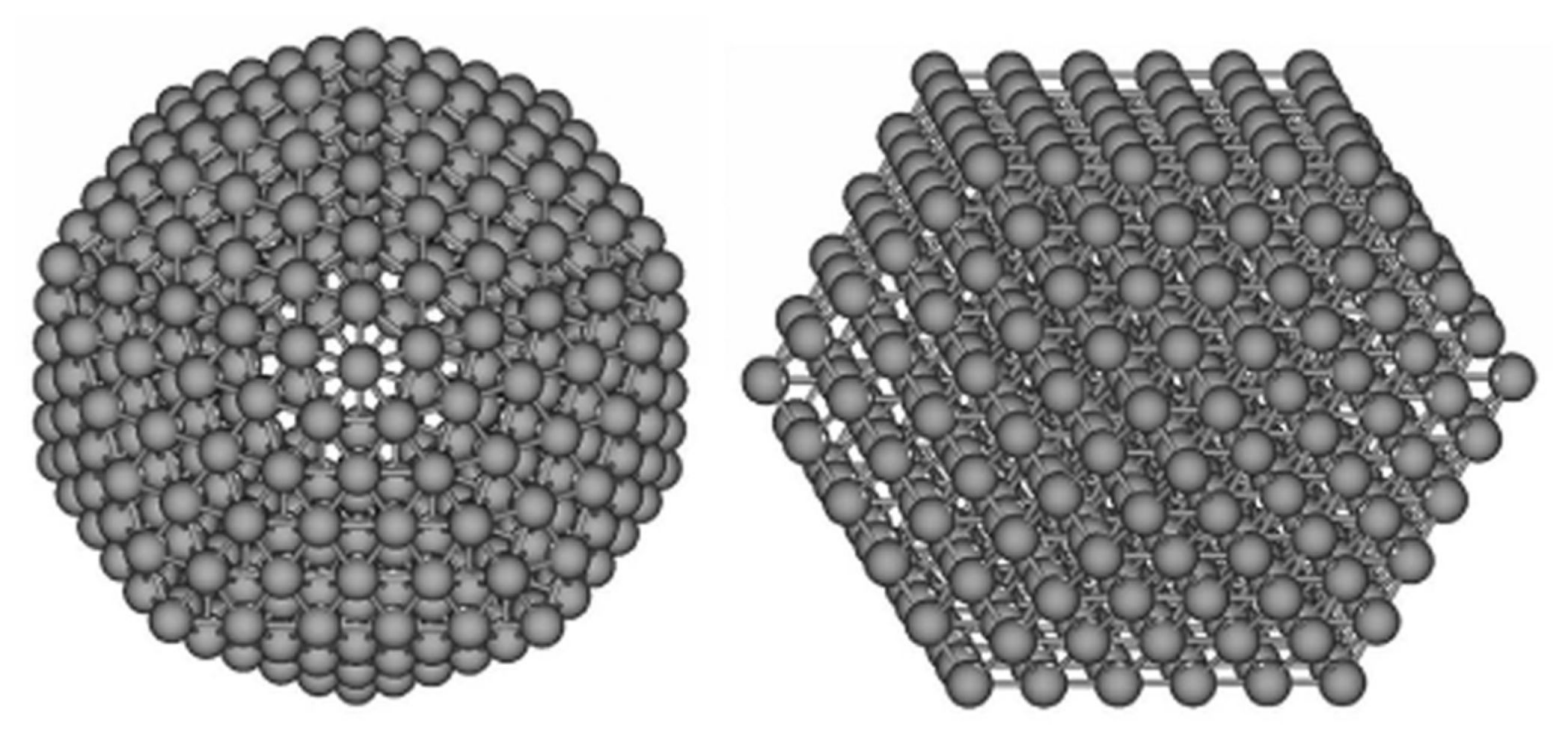
Icosahedral and cuboctahedral structures of Ag_561_.

**Figure 4:**
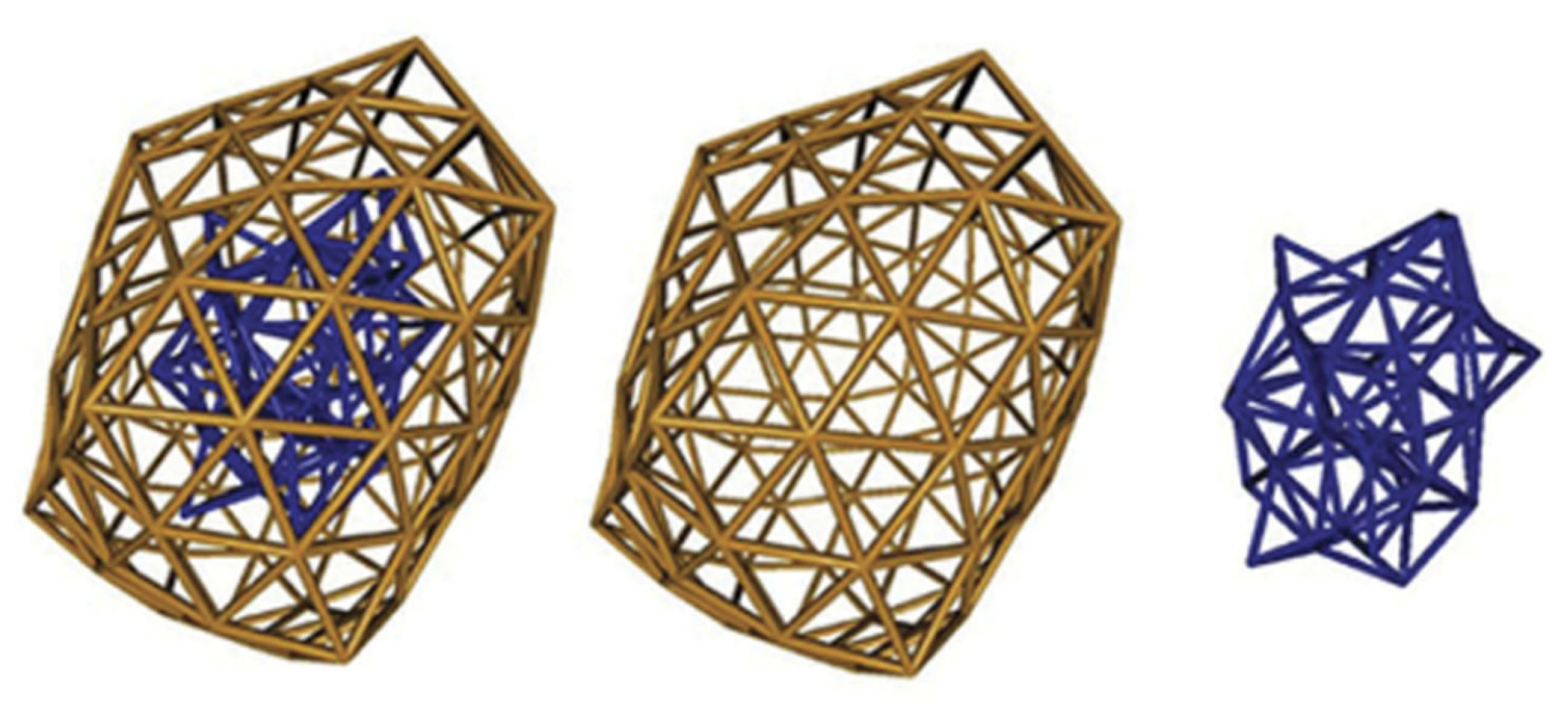
Core-shell like organization of the lowest energy Au_147_ isomer (left): surface atoms (middle) and core atoms (right) only. Adapted from reference [272] with the permission of AIP publishing.

DFTB has been used to investigate various clusters including sodium [262], ceria [295], cadmium sulfides [233,264], boron [166], silver and gold [155,157,165,172,173,267–272], ZnO [273], molybdenum disulfide [274], iron [154,275] or nanodiamond [276,277]. In addition to the necessary work dedicated to specific DFTB parametrization for these systems [155,156,172,173,268–270,278], a number of studies have been devoted to their structural characterisation [63,153,154,157,161,165,268,278]. Figure 3 illustrates examples of investigated structures for silver cluster Ag_561_ [172]. An interesting question is the evolution with size of the competition between ordered and disordered structures [157,165,173,272]. For instance, global exploration performed at the DFTB level followed by local optimization at the DFT level, suggested that Au_55_ presents cavities [173] (recently confirmed by two other DFT studies [63,279]), and showed that the amorphous forms of Au_147_ are expected to be more stable than the regular icosahedral ones, or at least very competitive with this latter at low temperature [272] (see Figure 4). Shi et al. evidenced the presence of a core/shell structuration in Pt-Ru alloys [155].

Let us also mention the original approach based on machine learning to correlate the structure/morphology of silver NPs (with diameters up to 4.9 nm) and their electron transfer properties [280]. The magnetic properties of clusters have also been investigated evidencing strong changes with the number of atoms for small iron clusters [154,275].

In addition to atomic clusters, molecular clusters have also been investigated within the DFTB framework. This requires to go beyond simple second order DFTB for a proper treatment of intermolecular interactions including various corrections as describe in section 3. The characterisation of the most stable structures for water clusters provide a picture of the isomer excitation spectra strongly depending on the number of molecules. The ordering found for those isomers with DFTB turn out to be essentially correct. For instance in the DFTB studies of Simon et al., the most stable water octamer is a cube, the next isomer lying 20 kJ.mol^−1^ above, whereas the most stable hexamer is a prism followed by 4 other isomers within 9 kJ/mol [281,282]. Interestingly, this structural size dependence induces different thermodynamic behaviors with higher melting temperatures for the octamer than for the hexamer (180K vs 80K [181]). The evolution of water clusters IR spectra with temperature was also investigated [282].

**Figure 5:**
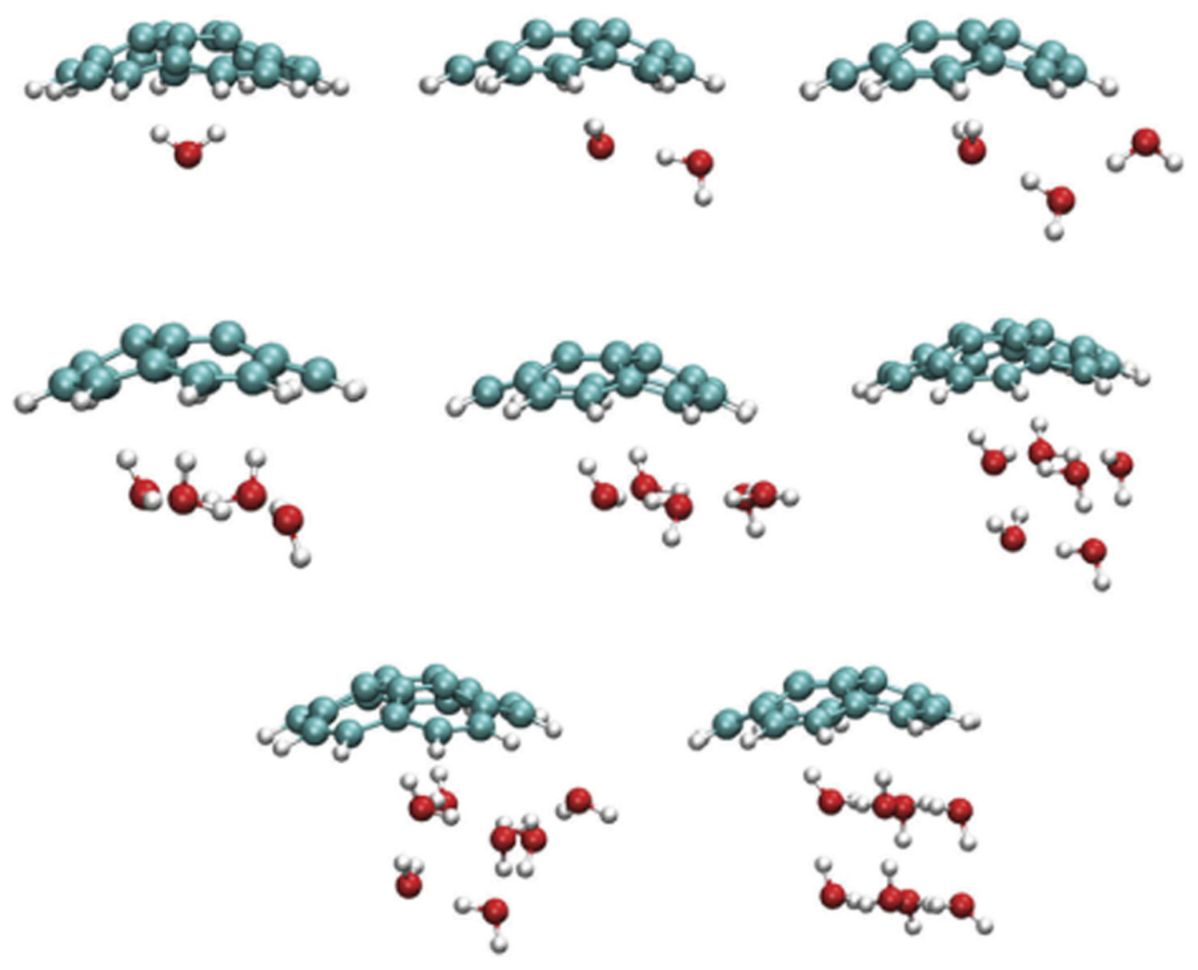
Most stable structures of C_20_H_10_-(H_2_O)_*n*_ (n = 1–8) obtained after PTMD/DFTB and local DFTB optimization following the procedure detailed in ref [96].

Understanding the interactions between water clusters and molecules is of prime interest as it can be regarded as a step towards the understanding of solvation. Besides, the interaction of water clusters with carbonaceous particles, and in particular polycyclic aromatic hydrocarbons (PAHs), has sustained a lot of interest lately due to their relevance in both atmospherical science and astrochemistry. The PES of water clusters in interaction with planar PAHs was explored with MD [281,282] and PTMD [181] simulations. The lowest energy structures of PAH-(H_2_O)_*n*_ clusters were determined for planar PAHs [281–283]. Figure 5 reports the lowest energy structures of corannulene (non planar PAH) in interaction with small water clusters C_20_H_10_-(H_2_O)_*n*_ (n = 1–8) obtained after PTMD simulations using a similar GO procedure as for C_16_H_10_-(H_2_O)_*n*_ clusters [96]. The interaction of the water clusters with the concave face of corannulene is the most energetically favorable, as previously shown for a single water molecule [284]. Interestingly, the water trimer tends to linearize, this is due to its interaction with the edge hydrogens, and such an effect is due to the finite-size of the systems [281,283]. Finite-temperature conformational dynamics of water clusters adsorbed on PAH were also studied [281,282] as well as the influence of PAH adsorption on the IR spectra of water clusters [281–283] and on their thermodynamic properties (heat capacities) [181].

Water clusters containing impurities, such as ammonium [167] or hydroxyde group [162] have also been considered within DFTB. New isomers were reported in the case of sulfate containing clusters (H_2_O)S${\text{O}}_{4}^{2-}$ and (H_2_O)H_2_S${\text{O}}_{4}^{2-}$ [174]. The application of DFTB to model protonated water clusters was first reported by Goyal et al. [285]. Korchagina et al. [175] showed that the cluster H_2_O 21H^+^ is particularly stable, in agreement with reference calculations [286,287], and present a specific behavior of the heat capacity curves also observed experimentally. The main differences between the IR spectra of pure and protonated water clusters have also been studied [288].

**Figure 6:**
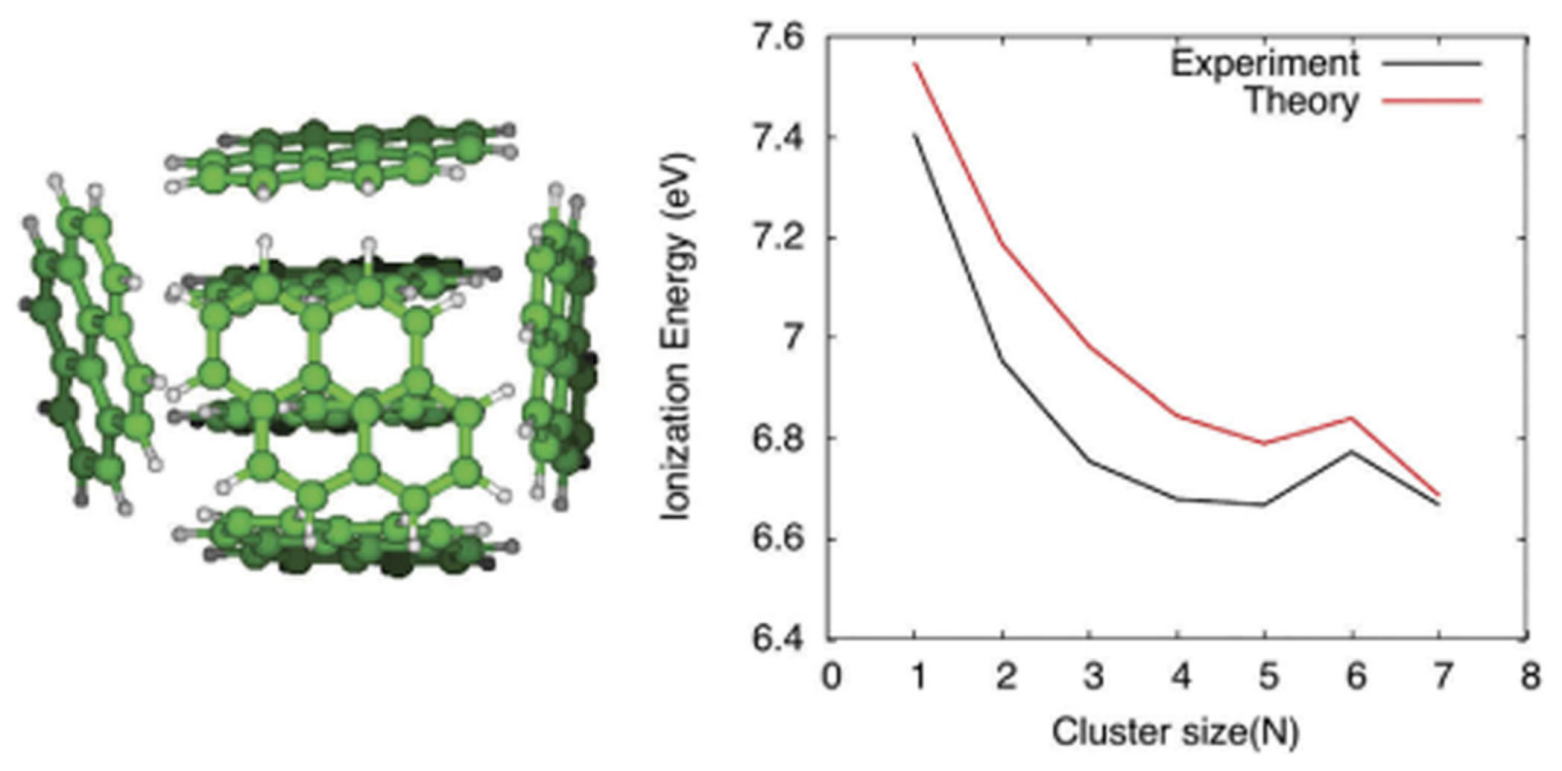
Left: DFTB most stable structure of the cationic pyrene heptamer with all-atom relaxation [150]. Right: experimental and computed ionization potentials for pyrene clusters. Adapted from reference [292] (https://doi.org/10/1021/acs.jpclett.7b01546, further permissions should be directed to the ACS).

When molecular clusters are singly ionized, alternative DFTB-CI schemes (see section 3.3) may be considered to properly describe the charge and excitation resonance over the different units. Its combination with global exploration schemes allowed to identify the most stable structures of cationic pyrene (Py) clusters, showing that the charge is delocalised over a dimer or trimer core [150], and to compute their electronic spectra [149]. This model was further used to interpret various experiments concerned with thermal evaporation of ${\text{Py}}_{2}^{+}$ clusters [289], photodissociation of ${\text{Py}}_{2}^{+}$ [290], combined photoionisation and dissociation of Py_2_ [291] and the determination of Py_n_ ionisation potentials (see Figure 6) [292].

Finally, let us note that the ability of DFTB for describing ionic clusters (clusters of ion pairs) and nanoparticles has recently been reported [293,294].

### 4.4 Functionalized clusters

The accuracy of the DFTB approach to model bare metal systems, inorganic particles [295–297] as well as organic molecules [205,209] combined with the transferability of the DFTB potentiel over different chemical systems, makes it a valuable tool to describe functionalized clusters and hybrid organic-inorganic systems. Hence, over the last 15 years, this strength of the DFTB approach has led to a number of studies devoted to functionalized clusters.

A number of them focused on metal particles, in particular gold and silver. In the case of gold, the study of thiolates has been of utmost importance as they are often used to stabilise gold particles. In this context, attachment of thiolates on gold clusters were first studied at the DFTB level by Mäkinen et al. [298]. The authors first validated the DFTB approach against experimental and DFT data for three systems: ${\text{Au}}_{25}{\left(\text{SMe}\right)}_{18}^{-}$, Au_102_ (SMe)_44_ and Au_144_(SMe)_60_ and on Au_102_(p-MBA)_44_ (p-MBA = paramercaptobenzoic acid). Then, they demonstrated its ability to accurately describe the low-energy structures of Au_m_(SMe)_n_ species as well as qualitatively describe their electronic structure. A similar study was latter conducted by Fihey et al. who developed a new set of DFTB parameters for AuX (X = Au, H, C, S, N, O) elements in order to better describe the interaction of thiolates and other molecules with gold particles [269]. Those parameters were validated by considering two species: Au_3_SCH_3_ and Au_25_SCH_3_ for which structural, energetic and electronic properties were calculated and compared to DFT results. Castro et al. also applied the DFTB approach to describe amino-acids grafted on gold clusters [299]. As for thiolathes, DFTB leads to geometries and adsorption energies that are in good agreement with DFT results, which allowed the authors to study the electron-acceptor and electron-donor character of several amino-acids grafted to gold clusters. In the case of silver, an elegant application of DFTB was conducted by Douglas-Gallardo et al. who tried to rationalize the impact of two adsorbates, water and 1,4-benzoquinone, on the surface plasmon resonance (SPR) band of silver particles of various sizes [300]. This study was a continuation of a previous work devoted to bare icosahedral silver nanoparticles undergoing strong laser pulses [301]. The characteristic of this SPR band, in particular excitation energy and line width, are key in the application of plasmonic particles. However, experiments can have difficulties in probing such properties as they strongly depend on size [302,303], morphology [302–304] and chemical environment [302] of the particles. Combining real-time excited-state dynamics and DFTB, Douglas-Gallardo and coworkers were able to draw a linear relationship between the surface plasmon excitation energy and the inverse cube root of the cluster number of atoms as well as the impact of the adsorbate molecule by studying five different cluster sizes: Ag_55_, Ag_147_, Ag_309_, Ag_561_ and Ag_923_. In a similar spirit, using real-time excited-state dynamics and DFTB, part of these authors also studied the impact of oxidation on the plasmonic properties of aluminum nanoclusters [305]. To do so, they first simulate the optical absorption spectra of five bare icosahedral aluminum nanostructures: Al_55_, Al_147_, Al_309_, Al_561_ and Al_923_. Then, focusing on Al_561_, MD simulation were performed to describe the structure of Al_561_ at different stage of oxidation, from which absorption spectra were re-evaluated. The resulting SPR band displays a red-shift, a broadening and a decrease in intensity that get stronger as oxidation state increases. This was shown to result from the presence of oxygen and not from the symmetry loss.

**Figure 7:**
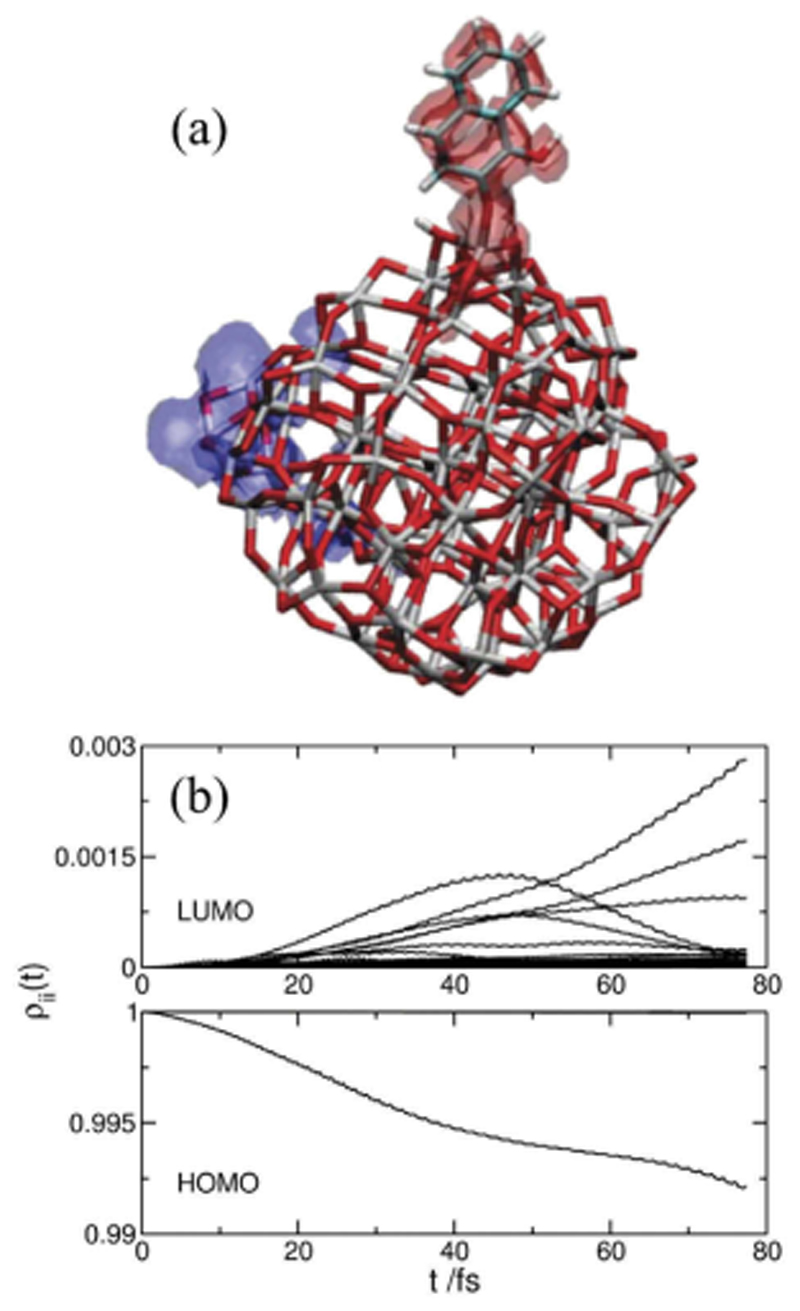
(a) Schematic representation of the atomic structure of a naphthalenediol-TiO_2_ complex. Superimposed are the corresponding HOMO (red) and LUMO (blue). (b) Timedependent population of the HOMO and higher-energy orbitals for naphthalenediol-TiO_2_ subject to a continuous laser-type perturbation. Naphthalenediol-TiO_2_ undergoes a direct injection mechanism where population exchange occurs between the HOMO and a manifold of high-energy orbitals. Adapted with permission from [306]. Copyright (2012) American Chemical Society.

DFTB has also been applied to model the behavior of dyes grafted on inorganic particles, mainly TiO_2_, under light excitation to understand charge injection mechanisms in dye-sensitized solar cells (DSSC) [306– 308]. Indeed, in Grätzel-type solar cells, photoexcitation of the grafted dyes leads to the injection of electrons into the conduction band of the semiconductor. Understanding this mechanism is thus a key step in developing more efficient DSSC. To provide an atomistic-scale description of this process, electron photoinjection was described at the DFTB level for various dyes: alizarin, coumarin C343, derivatives of aniline, naphthalenediol [306], catechol, cresol [307] on a TiO_2_ cluster and 4-nitrophenyl-acetylacetonate and coumarin 343 on a polyoxotitanate particle [308]. Note that Fuertes et al. also studied at the DFTB level the optical properties of bare TiO_2_ particles [266]. These various studies allowed to understand the different steps of the electron transfer from the dye to the inorganic particles for both type I and type II mechanisms and the influence of the excitation wavelength. As a representative example, Figure 7 shows how the electronic structure of a naphthalenediol-TiO_2_ system evolves when subject to a lasertype perturbation. The population exchange between the HOMO and an excited state of the dye followed by an electron transfer to the conduction band of the semiconductor is characteristic of a indirect injection mechanism as opposed to a direct mechanism where the exchange directly occurs from the HOMO of the dye to the semiconductor conduction band [306].

### 4.5 Supported or embedded systems

**Figure 8:**
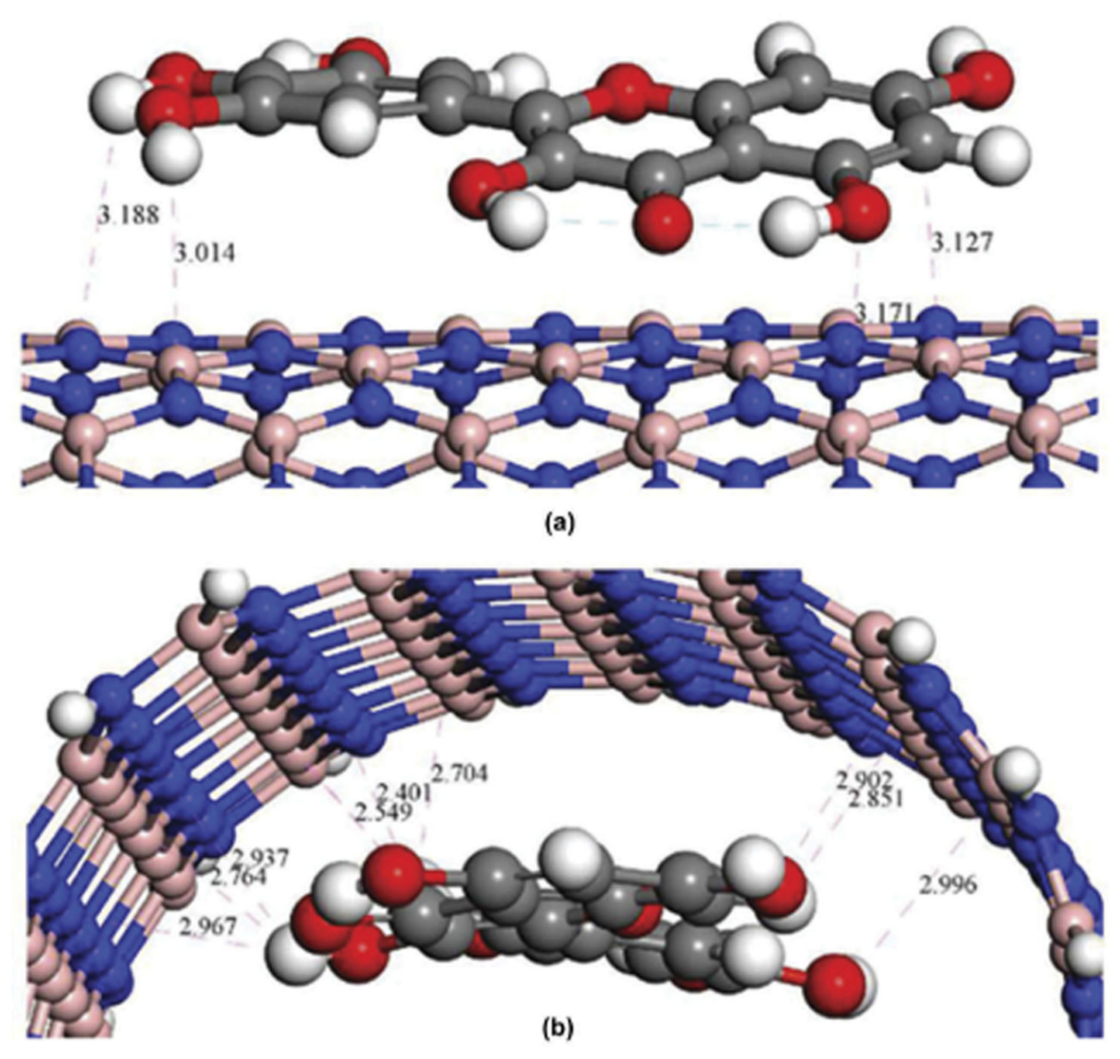
The near site view of the flavonols outside (A) and inside (B) the BNNT surface. The closest contact distance is also shown. Reproduced from [315] with permission of John Wiley and Sons.

DFTB has been widely used to study the adsorption of organic molecules on oxide surfaces. First of all, the adsorption of small molecules such as CO_2_ or NH_3_ on ZnO was studied and the results were found to be in agreement with both DFT and experimental data [296]. Subsequently, the adsorption modes of larger molecules were studied. The grafting of a zwitterionic amino acid (glycine) on a germinal hydroxylated silica surface showed a domination of the adsorption through the carboxylic acid group vs the ${\text{NH}}_{3}^{+}$ one in an explicit water environment [309]. The effect of water has been investigated in the case of the adsorption on TiO_2_ of a serine molecule, an amino acid slightly larger than glycine. It was found that the presence of water weakens the O-Ti bonds and H-bonds existing between the -COO^−^/-OH groups and the surface [310]. The effect of the grafting of an organic molecule on the surface gap has also been explored and was found to be negligible in the case of an acetic acid molecule adsorbed either on a crystalline oxide surface (anatase (101), rutile (110) and (B)-TiO_2_ (001)) or on an amorphous one ((a)-TiO_2_) [311]. More recently, the development of new DFTB parameters has also made it possible to study the adsorption of organic molecules on metal surfaces. One can for example mention a study of the adsorption of a corrosion inhibitor (chalcone derivative) on a Fe(110) surface in which the π molecular orbitals were found to play a major role in the adsorption phenomenon [312]. DFTB was also developed in order to study adsorption of organic molecules on carbon surfaces, for example transition metal complexes (porphyrin and porphycene) on graphene [313] or small molecules (H_2_O, CH_4_, NH_3_) on defective carbon nanotubes which were all found to physisorb on the nanotubes, except NH_3_ which also chemisorbs [314]. Optical properties of natural pigments (flavonols) adsorbed on boron nitride nanotubes were also analyzed using DFTB (Figure 8) [315]. Some DFTB surface adsorption studies have also given rise to reactivity studies, for example water splitting on anatase (001) [316] or H_2_ dissociation on plutonium [317].

The adsorption of a PAH on a water ice surface and its influence on the PAH properties are relevant topics for interstellar chemistry. In dense molecular clouds, PAHs are likely to condense on grains covered by H_2_O rich ice mantles with exposure to ionizing radiation [318], and a rich heterogeneous photochemistry on interstellar grains is expected to occur [319]. This motivated experimental studies where PAHs in an icy environment are irradiated with UV-photons leading to the following statements;-(i)-the interaction with the ice leads to a decrease of the ionisation energy of the PAH by 1.5 to 2 eV [320,321] and (ii)the photo-initiated reactions of PAHs with water on the ice surface [322,323], even at low energy, could be ion-mediated [324]. In this context, Michoulier et al. [96] determined the effect of ice on the ionization energies (IEs) of PAHs using DFTB and constrained DFTB schemes [96] for a series of PAHs from naphthalene (C_10_H_8_) to ovalene (C_32_H_14_) on different types of ices, crystalline (hexagonal Ih and cubic Ic) and amorphous (low density amorphous LDA). They also observed a correlation between the presence (resp. absence) of dangling OH (dOH) bonds interacting with the PAH and the increase (resp. decrease) of the PAH ionisation energy [96]. The conclusion is that the small magnitude of the IE variation, that is at most 0.8 eV for amorphous ice (the experimental type of ice) cannot account for the experimental results. Actually, the electron ejected from the PAH could be transfered to the water ice or recombine with impurities such as the OH radicals. A future theoretical challenge will be to treat such an electron transfer process.

Furthermore, in the astrophysical context, the IR signature of the adsorption of PAH on water ice is an issue of paramount relevance with the imminent launch of the James Webb Space Telescope, which will aim at providing high resolution IR spectra from various regions of the interstellar medium. Therefore, diagnostics for the presence of PAHs condensed on water ice need to be established beforehand. Using the efficiency of DFTB, combinations of harmonic IR spectra of several PAH-amorphous ice systems possessing various PAH-surface interacting structures was computed. The shifts of the dOH bond frequencies induced by the adsorption of the PAH were found to range from −70 to −85 cm^−1^ depending on the PAH, in good agreement with experimental results [325]. Further details about the description of water based systems with DFTB can be found in a previous review [326].

Beyond the adsorption of single molecules, the DFTB method, due to its low computational cost, also allows for the study of extended monolayers. In this framework, the impact of an organic molecule layer on the tunneling current was studied in the case of a PTCDA (3,4,9,9,10-perylene tetracarboxylic dianhydride) monolayer on a 2 × 1 S-passivated GaAs (100) surface. The presence of the layer was found to reduce by one order of magnitude the current with respect to the free surface, in agreement with experimental data [327]. Monolayers (OH, HS and S) were also added in a DFTB study of the sulfidization-amine flotation mechanism of smithsonite in order to model the hydration effect of water and the sulfidization effect on the ZnCO_3_ (101) surface [328]. The structural study of a water monolayer on oxide surfaces has led to several DFTB studies, for example on ZnO [329], on a TiO_2_ anatase surface [330] or on an alumina surface on which it has been found that water dissociates rapidly, leading to an -OH group coverage of about 4.2 groups/nm^2^ [331]. Finally, one can also find a DFTB study of graphene formation on a surface-molten copper surface. In the latter, the authors explains the high quality of a graphene layer grown on Cu by the fact that the high mobility and rapid diffusion of surface Cu atoms induce defecthealing during graphene growth [332].

The deposition of clusters on surfaces has also led to a few studies at the DFTB level. Structural and energetic changes were reported when potassium clusters up to 20 atoms adsorb on a potassium surface K(110) or K (100) [333], the interaction energy being found to dominate the structural reorganization one. MgO supported Au islands were also studied [334,335]. In these islands, the inner atoms were found to remain neutral while the perimeter ones were found to be negatively charged. The specific role played by the peripheral atoms during adsorption and reaction processes was attributed to this charge accumulation coupled with a high density of state.

**Figure 9:**
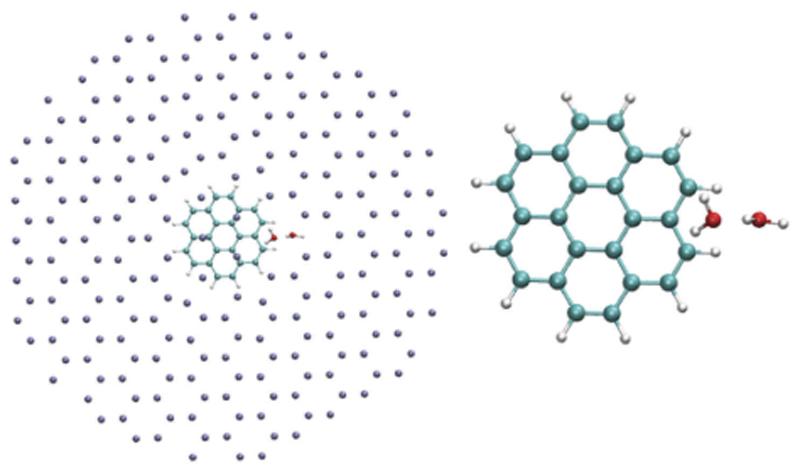
Structure of a water dimer interacting with coronene within an argon rare gas matrix subpiece treated *via* a DFTB-MM scheme [335].

Finally, structural properties and IR spectroscopy of carbonaceous molecules, water molecules and complexes embedded in cryogenic argon matrix were investigated *via* the DFTB-MM model described in section 3.5 [70,131,335]. The structuration of a water dimer/coronene complex within the argon matrix is illustrated in Figure 9. Fine effects such as the modification of the energetic order of the (H_2_O)_6_ isomers with respect to the gas phase were shown. Besides, MD simulations using the DFTB-MM model allowed to show the influence of (even low) temperature (10 K) on the IR spectrum of a single water molecule embedded in the Ar matrix: red shifts and broadening experimentally observed with respect to the gas phase could be interpreted [335].

### 4.6 Vibrational spectroscopy

Determining theoretical vibrational spectra of large systems is an important issue as such spectra are among the most popular diagnostics for the presence of species in laboratory experiments, in the earth atmosphere or in space. The determination of vibrational spectra requires the description of charge fluctuation. The use of DFTB2 (possibly with extensions) or DFTB3 thus appears as a convenient approach to compute the vibrational spectra of large molecular systems or clusters as well as the anharmonic effects due to the PES on the spectra.

IR or Raman vibrational spectra can be modeled in the double harmonic approximation. The normal modes are obtained by diagonalizing the full weighted hessian matrix while intensities are obtained by evaluating the variations of the dipole moments (IR) [336] or the changes of the molecular polarizability tensor (Raman) induced by the normal mode oscillations [337]. Vibrational spectra at the DFTB2 level were benchmarked on small molecules with respect to hybrid DFT methods in particular [338], showing that the approach could be used to compute the vibrational spectra of large organic molecules. For instance, the structures of the isomers of oxidized graphene nanoflakes were differentiated by their IR spectra and a correlation was established between stability and IR data [339]. The IR spectra of carbon clusters, either individual structures [227–230,340], or populations (families of isomers) of astrophysical interest [232] have also been determined.

When the internal energy increases or/and when systems exhibit a floppy behaviour, as for instance molecular clusters or systems of biological interest, anharmonic effects due to the shape of the PES are likely to become non negligible. Anharmonic effects on vibrational spectra can be obtained from on-the-fly MD computing the time correlation function of the dipole moment (IR) or of the polarizability (Raman) [341]. The DFTB approach is convenient because long simulations are possible and convergence of spectra in terms of positions and intensities can be reached in reasonable computational time (ns scale) for systems of several tens of atoms [177]. This approach allows to describe the expected redshift of the modes (when no coupling occurs). The example of the out-of-plane CH mode (*γ*_*CH*_) of PAHs is quite illustrative. A linear fit of the shift of the latter mode as a function of the internal energy (kinetic temperature) yields the anharmonicity coefficient, the value of which determined at the DFTB level was comparable to the experimental one [342]. This approach was applied to complexes of astrophysical relevance such as SiPAH and FePAH, for which increasing the energy leads to an enhanced motion of the atom (Si, Fe) on the PAH surface [221,342]. In the case of Si, this leads to a merging of the *γ*_*CH*_ modes, that are resonant at two different energies at low temperature and thus induce a deviation from linearity of the function ν_*γ*_*CH*__ (*T*) [342]. Using the same approach, it was found that the influence of the coordination of water clusters on PAHs led to a modification of the anharmonicity of the *γ*_*CH*_ mode, and that this could be a fingerprint of the edge-coordination of the water cluster on the PAH [283]. In the case of a water molecule (described at the DFTB level) surrounded by a rare gas matrix (described with a force field FF), it was shown from MD// DFTB/FF simulations that at low temperature (∼10 K), the water molecule rotates inside the matrix (in agreement with experimental results at low concentration of water), and that leads to red shifts and broadening of the water stretching modes [335] (for a review, see ref [326]).

### 4.7 Reactivity and fragmentation

**Figure 10:**
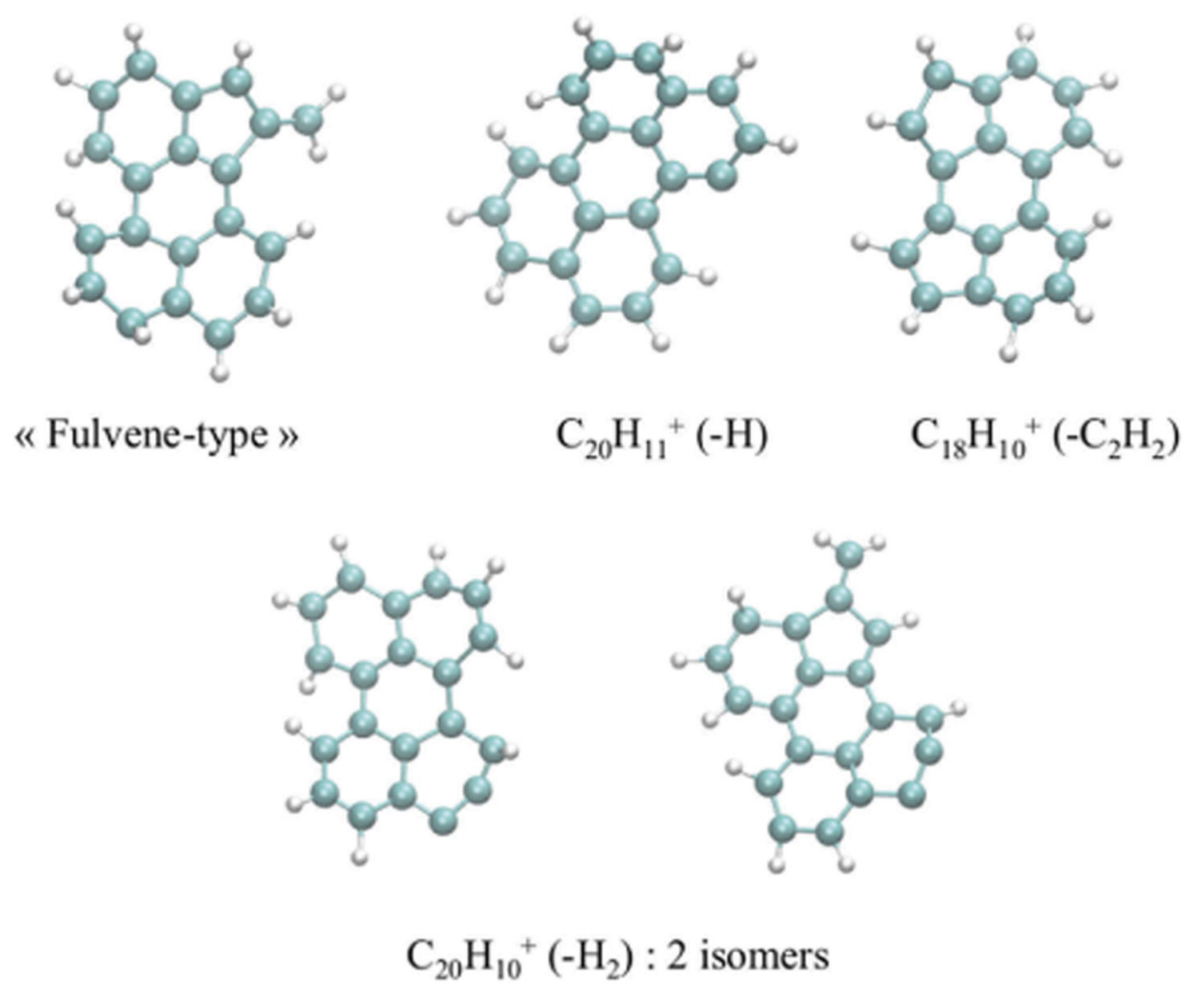
Snapshots retrieved from MD/DFTB simulations describing the evolution of cationic perylene [C_20_H_12_]^+^ at high energy (∼24–26 eV of internal energy): the formation of a fulvenetype isomer was observed, as well as losses of H, H_2_ and C_2_H_2_, the expected statistical dissociation pathways for PAH radical cations [346].

The efficiency of DFTB allows for dynamical reactivity studies that can be achieved either through MD/DFTB simulations or through biased molecular dynamics techniques [343] such as umbrella sampling [344] and metadynamics [345]. Statistical convergence on averaged properties can be reached taking into account explicitly the electronic structure for quite large systems. We can cite for instance the unimolecular reactivity of isolated molecular systems in the gas phase such as the isomerisation [177] and dissociation at high energy [346–348] of PAH radical cations. MD/DFTB simulations provide insights into statistical dissociation branching ratios and pathways. The competition between isomerisation and dissociation was shown (see as an illustrative example some isomers and cationic fragments structures of cationic perylene [C_20_H_12_]^+^ in Figure 10). Comparison with experimental results reporting collision induced dissociation of PAHs [348] or competition between hydrogenation and dissociation of PAHs [347] gave satisfactory results and allowed to cross-benchmark the approaches.

The low energy conformational dynamics of water clusters, isolated and adsorbed onto a molecular PAH was addressed [181,281,282]. Bimolecular reactions were also investigated *via* collision dynamics simulations, for instance the collision of H with CO adsorbed on water clusters [349] or the hydrogen uptake of carbon fullerene cages and boron doped heterofullerene [350]. Finally, MD/DFTB simulations at high temperature in simulation chambers were performed to study the growth of carbonaceous systems: formation of large carbonaceous species with various structural orders formed from mixtures of benzene varying the H/C ratio [351–353], growth of carbon nanotubes, possibly catalyzed by a metallic clusters (iron [354,355]), on a SiC surface [356], or formation of metallofullerenes [357].

### 4.8 Thermodynamics

Some studies have been concerned with the evolution of structural properties with temperature, as well as the determination of the heat capacities of clusters, taking advantage of Parallel Tempering strategies. For instance Choi et al. [163]. simulated the caloric curve of the water octamer. Note that although the qualitative evolution is expected to be well reproduced, one should keep in mind that the simulation results may depend on the type of DFTB and parametrization used [163,358]. A subsequent work was published by Oliveira et al. who redetermined the caloric curves of the water hexamer and heptamer [181]. They also investigated in details the microscopic nature of the phase transition at melting, fingerprinting in particular the evolution of the isomer populations. They furthermore investigated the effect of depositing water clusters on a graphite type substrate modeled as a coronene molecule. Other DFTB thermodynamical studies were concerned with metallic systems and in particular silver and gold clusters. The effect of charge on the doubly magic (electronically closed shell and geometrically a symmetric pyramid) cluster Au_20_ was investigated [359] as well as the the correlation between the isomer spectra features and the nature of the solid-to-liquid transition [360], from the comparison between the caloric curves of structurally ordered systems (Au_20_, Ag_55_) and those of disordered cases (Ag_20_, Au_55_).

### 4.9 Dynamics in excited states

**Figure 11:**
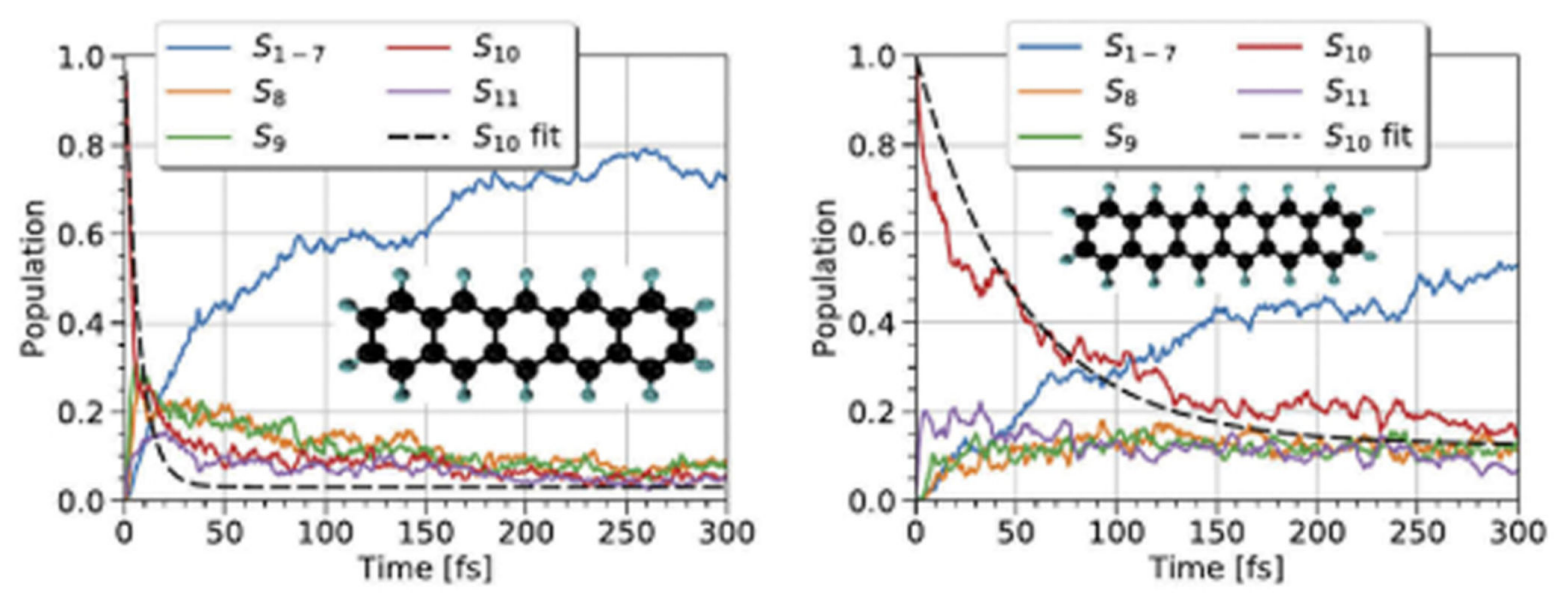
Example of FSSH molecular dynamics simulation for neutral polyacenes [203]. Population dynamics averaged over 63 trajectories following excitation to the brightest excited *S*_10_ state in pentacene (left panel) and hexacene (right panel). Adapted by permission of the PCCP owner societies.

The TD-DFTB method was successfully used to study the charge migration in the caffeine molecule induced by an ionizing XUV pulse [361]. In addition to the simulation of exciton dynamics in molecular clusters [200,362–364] reported in section 4.3, the FSSH scheme for non-adiabatic dynamics has been used to simulate excimer formation in the pyrene dimer [365] or relaxation of excited fluorene oligomers [200]. Relaxation dynamics enhanced by transition density analysis has been investigated by Stojanovic et al. for two cycloparaphenylene molecules (labelled [8]CPP and [10]CPP) in ref [201]). Other authors have studied the intraband electron and hole relaxation as well as nonradiative electron-hole recombination in a CdSe quantum dot and the (10,5) semiconducting carbon nanotube [202]. The version of FSSH coupled to TD-DFTB in the DeMonNano code was used to investigate the relaxation mechanisms in neutral polyacenes (see Figure 11) ranging in size from naphthalene to heptacene, showing an alternation in decay times of the brightest singlet state with the number of aromatic cycles. More details about the implementation as well as discussion about the observed size effect can be found in ref [203].

Electronic excited states of molecular clusters have also been investigated via DFTB-based schemes. The excitation energy transfer in molecular aggregates has been described through a Frenkel Hamiltonian whose parameters are computed from TD-DFTB [147,148,366,367]. The combination of non-adiabatic dynamics with long-range corrected DFTB [200] has been used to simulate the dynamical evolution of excitons in clusters of tetracene [362] and perylene diimides [363]. The dynamical coupling between local and charge transfer excitons in pentacene clusters was also investigated [364].

Another promising application of DFTB for large metal NPs concerns plasmonics [300,301,368]. For instance, the sub-picosecond breathing-like radial oscillations following a laser pulse excitation have been evidenced for silver NPs up to 309 atoms [301].

## 5 Outlines and perspectives

The Density Functional based Tight Binding Theory is now more than 25 years old. With respect to many other usual Tight Binding theories, it displays several advantages. One is that it is based on a formal expansion of the energy as a function of the density. Thus, it can be expanded and improved by considering significant terms at higher orders of the expansions, which provides a theoretical basis for upgrade. Being derived from DFT, DFTB exhibits the drawbacks inherent to the former, such as being practically a mean field theory since the exact exchange-correlation functional remains unknown, or suffering from self-interaction errors. In the same time, it has also benefited from many methodological developments adapted from DFT, such as the long-range/short- range separation scheme or the time-dependent version which provides access to excited states, visible/UV spectra and non-adiabatic dynamics. Important initial weaknesses, such as poor treatment of non-covalent interactions, have been cured through various complementary schemes.

DFTB has been now implemented in several packages such as DFTB+ [369], DeMonNano [370], ADF [371], Amber [372], Gromacs [373], Gaussian [374], DFTBaby [200], CP2K [375] where various functionalities are available. Parameters are now available for a large set of elements, even though the problem of the determination and transferability of the repulsive form must still undergo further progress. Of course, many applications have been made for standard atoms C, H, O, N, P, Si, etc. for which the transferability of various DFTB parameter sets has been tested, possibly combined with various versions of DFTB. For other elements, for instance transition metals or even heavier elements, transferability is still to be fully assessed. Machine learning might be useful to finalize the parametrization work [64].

In the domain of DFTB-MM methodologies, combination of DFTB with polarizable force-fields for liquids, and in particular water, would certainly yield a desirable advance for molecules in liquid phase, and even for chemistry with ice. Multi-spatial shell treatments (the active system and a near shell of water molecules treated explicitly with DFTB, the other ones addressed *via* accurate polarizable force fields) may also improve the study of reactivity in cases where the solvent is likely to participate in the process.

With the development of TD-DFTB and related formalisms, photochemistry and electron transfer processes become feasible for quite large systems. In the field of excited states, an obvious lack concerns Rydberg states which cannot be reached in DFTB, based on valence orbitals only. It could be interesting to include diffuse basis functions that would make at least the low Rydberg states available. Also, DFTB is based on a LCAO expansion and is thus a theory for bound states. As in LCAO-based methods, the continuum is only poorly represented by a discrete set of virtual orbitals, even worse with DFTB. Development of matrix coupling to the continuum could make it able to describe molecular physics processes involving unbound electrons (ionization, electronic attachment).

Many important processes involving light atoms require a quantum description of nuclei motion, for instance flexible molecules, reactions associated with proton transfer, water dynamics and ice dynamics. Implementation of quantum dynamics of nuclei *via* the Path Integral Molecular Dynamics (PIMD) with DFTB electronic structure was reported recently [377]. PIMD yields a system of replicas which multiplies the actual number of degrees of freedom by a factor between 8 and 32, depending on the target accuracy. Development of PIMD within the DFTB framework for highly parallel computing architectures should make nuclei quantum dynamics affordable even for rather large and complex systems in gas phase.

Despite the fact that the present paper is essentially devoted to finite systems, it is important to mention that DFTB in various distributions is implemented in periodic version to address crystals and condensed matter. DFTB offers the possibility to achieve bulk matter simulations using large unit cells (above 10^3^ atoms). This can be of primary importance for investigating the dynamics of default propagation in pure metal and alloys at the microscopic scale. The detailed interaction of atoms, molecules or clusters with surfaces can also be investigated *via* DFTB within the periodic framework. Deposition of clusters on surfaces may drastically change their structural, spectroscopic, chemical or thermodynamical properties. Such studies also lead to the conception of nanodevices including nanostructuration, nanowires, nanotransport [194]. A neighbouring topic is the collision of atoms or molecules with metal surfaces which may exhibit quite complex electron-surface dynamical coupling involving phonons, plamons and holeelectron pairs excitation. Such complex physics can be addressed by DFTB considering explicitly all the atoms of the active systems and of the surface slabs. Methodological developments can also be thought by combining classical phenomenological description accounting for electron-pair excitation and DFTB *via* a dissipative dynamics in the ground state [378].

**Figure 12:**
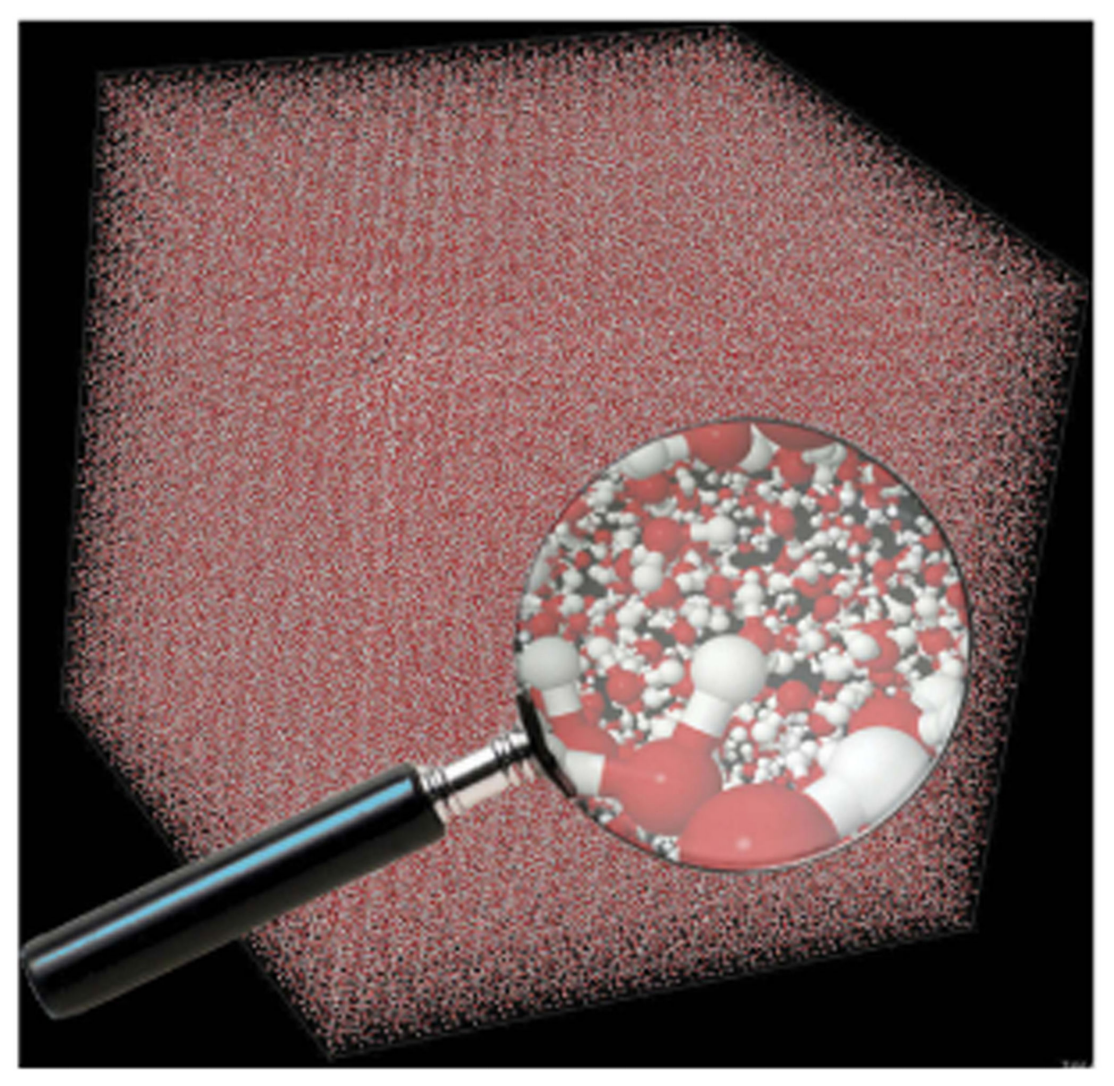
Box of 350000 water molecules treated *via* a DFTB cluster division algorithms [376]. Adapted with permission from (J. Chem. Theory Comput. 2014, 106, 2344–2354). Copyright (2014) American Chemical Society.

Finally, a word can be said about computational efficiency. Standard DFTB2 is 10^2^ to 10^3^ times faster than even local functionals, and even more if compared with higher-level functionals such as hybrid, double hybrid or LC-corrected functionals. Algorithmic schemes achieving linear scaling with the number of atoms in solving the DFTB Hamiltonian [21,376,379–382] such as the Divide and Conquer techniques [21,381,382] or cluster type algorithms [376] have now proved the feasibility of calculations on extremely large systems up to one million atoms at least for covalent or intermolecular complexes (see Figure 12: a box of 350000 water molecules), even though one should mention that the case of metals remains more delicate due to electronic delocalization. Even if large scale dynamical simulations on such huge systems are not yet practicable, DFTB certainly stands as a promising method to address simulations of systems with up to 10000 atoms on the next generation of High-Performance Computing architectures, which would be quite helpful for theoretical investigation of properties and processes involved in the chemistry and physics of large molecular systems, possibly biomolecules, or in nanoparticle physics.
